# Dose-Dependent Regulation of Alternative Splicing by MBNL Proteins Reveals Biomarkers for Myotonic Dystrophy

**DOI:** 10.1371/journal.pgen.1006316

**Published:** 2016-09-28

**Authors:** Stacey D. Wagner, Adam J. Struck, Riti Gupta, Dylan R. Farnsworth, Amy E. Mahady, Katy Eichinger, Charles A. Thornton, Eric T. Wang, J. Andrew Berglund

**Affiliations:** 1 Department of Chemistry and Biochemistry, Institute of Molecular Biology, University of Oregon, Eugene, Oregon, United States of America; 2 Department of Biology, Johns Hopkins University, Baltimore, Maryland, United States of America; 3 Department of Biology, Institute of Molecular Biology, University of Oregon, Eugene, Oregon, United States of America; 4 Department of Neurology, University of Rochester Medical Center, Rochester, New York, United States of America; 5 Department of Biology, Massachusetts Institute of Technology, Cambridge, Massachusetts, United States of America; 6 Koch Institute for Integrative Cancer Research, Massachusetts Institute of Technology, Cambridge, Massachusetts, United States of America; 7 Department of Biochemistry & Molecular Biology, Center for NeuroGenetics, University of Florida, Gainesville, Florida, United States of America; The Jackson Laboratory, UNITED STATES

## Abstract

Alternative splicing is a regulated process that results in expression of specific mRNA and protein isoforms. Alternative splicing factors determine the relative abundance of each isoform. Here we focus on MBNL1, a splicing factor misregulated in the disease myotonic dystrophy. By altering the concentration of MBNL1 in cells across a broad dynamic range, we show that different splicing events require different amounts of MBNL1 for half-maximal response, and respond more or less steeply to MBNL1. Motifs around MBNL1 exon 5 were studied to assess how *cis*-elements mediate the MBNL1 dose-dependent splicing response. A framework was developed to estimate MBNL concentration using splicing responses alone, validated in the cell-based model, and applied to myotonic dystrophy patient muscle. Using this framework, we evaluated the ability of individual and combinations of splicing events to predict functional MBNL concentration in human biopsies, as well as their performance as biomarkers to assay mild, moderate, and severe cases of DM.

## Author Summary

Our studies provide insight into the mechanisms of myotonic dystrophy, the most common adult form of muscular dystrophy. In this disease, a family of RNA binding proteins is sequestered by toxic RNA, which leads to mis-regulation and disease symptoms. We have created a cellular model with one of these family members to study how these RNA binding proteins function in the absence of the toxic RNA. In parallel, we analyzed transcriptomic data from over 50 individuals (44 affected by myotonic dystrophy) with a range of disease severity. The results from the transcriptomic data provide a rational approach to select biomarkers for clinical research and therapeutic trials.

## Introduction

Alternative splicing increases the coding potential of a gene and importantly, allows for regulation of expression of specific isoforms in a developmental and tissue-specific manner. Regulation of alternative splicing is integral for a variety of biological processes including erythropoiesis, neuronal differentiation, and embryonic stem cell programming [1,2]. Misregulation of alternative splicing alters isoform ratios and can cause cancers, muscular dystrophies, and neurological diseases [1–5]. Isoform ratios can be altered by differential expression of tissue specific factors [6,7]. Active areas of investigation in the alternative splicing field include how concentrations of alternative splicing factors affect isoform ratios, and what properties determine whether an isoform is responsive to a broad range of splicing factor concentrations or sensitive to a threshold level of activity.

To address these questions, we focused on alternative splicing regulation by MBNL1, an RNA binding protein involved in muscle, heart, and CNS development [8,9]. In the disease myotonic dystrophy, the activities of MBNL1 protein and its paralogs, MBNL2 and MBNL3, are reduced via sequestration to toxic RNAs, resulting in a titration of MBNL proteins away from their pre-mRNA substrates. MBNL proteins bind to YGCY motifs using Zn finger RNA binding motifs [10,11], including sequences found in the toxic RNAs (expanded CUG and CCUG repeats) that cause both myotonic dystrophy type 1 (DM1) and myotonic dystrophy type 2 (DM2) [12–16]. As has been observed in other microsatellite repeat diseases, the average length of the repeat in patients is loosely correlated with age of onset; repeat lengths also vary across cells and tissues within a single patient, potentially sequestering MBNL proteins to differing degrees [17]. MBNL splicing targets are differentially affected by the disease; DM1 patient samples exhibit a broad range of alternative exon inclusion levels, as compared to control samples containing low numbers of repeats [17–19], and MBNL1-dependent splicing events were shown to behave differently in response to different doses of MBNL1 protein [20].

Tunable systems can be used to control expression of specific genes and they can be used to produce a range of mRNA and protein isoforms, and phenotypes that change gradually or sharply, in response to stimuli [21]. Here, we use a tunable system to demonstrate that splicing events are responsive to MBNL1 dose, and characterize their behavior over a range of MBNL1 protein levels. We found that dose-responsive behaviors differ in their steepness, and differ in the protein concentration required to reach half-maximal splicing activity. By further studying a splicing event that is highly conserved between species, we assessed the importance of MBNL1 cis-element organization in controlling dose-response behavior. Similar to the cell-based studies, analysis of muscle biopsies from DM1 patients revealed that splicing events are not perturbed uniformly; here we characterize those splicing events across patients and calculate the inferred MBNL dose, or predicted free MBNL protein concentration, in each patient. These studies lead to predictions for which alternative splicing events are the most robust disease biomarkers.

## Results

### A cell model with a broad concentration range of MBNL1

**Fig 1:**
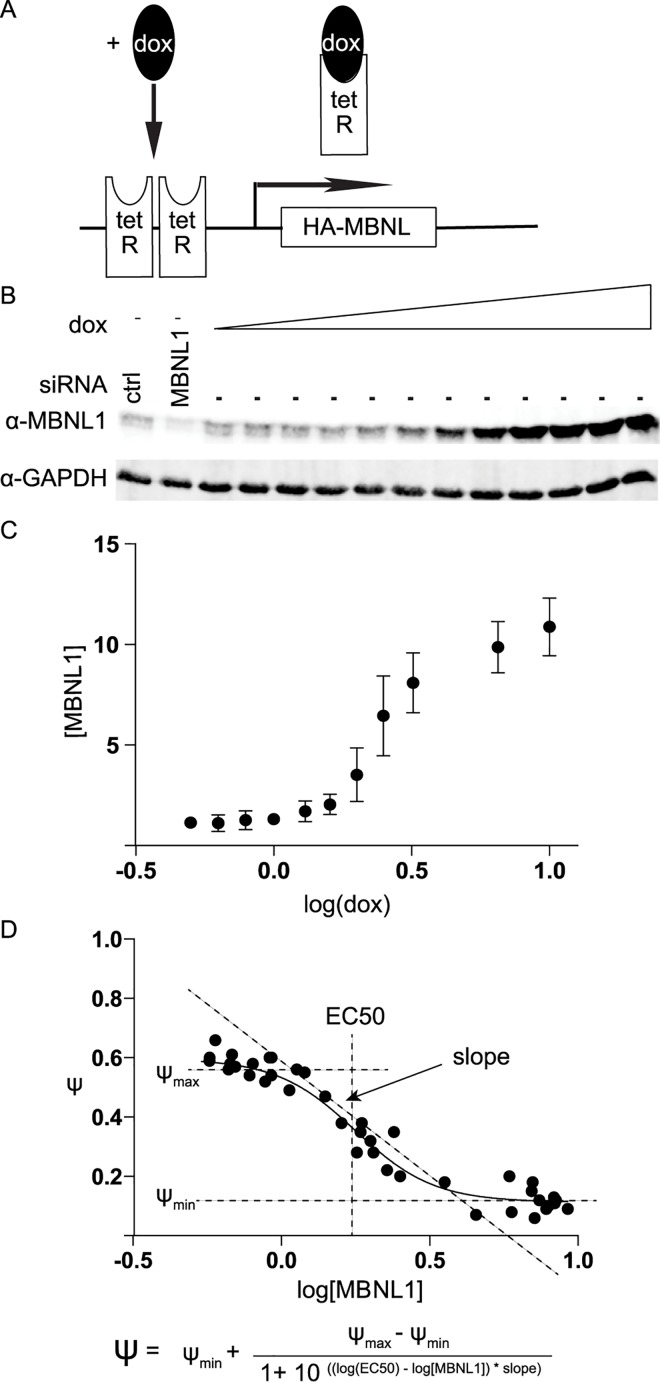
Titration of MBNL1 using an inducible tet-on system. (A) HEK293 cells were stably transfected to integrate a tetracycline inducible HA-MBNL1 expression construct into the genome. Addition of the tetracycline analog, doxycycline, de-represses inhibition of transcription by binding to the tet Repressor resulting in expression of full length HA-MBNL1 mRNA. (B) MBNL1 immuno-blot showing MBNL1 protein gradient resulting from dox (ng/ml) titrations. GAPDH serves as a loading control. The first lane represents treatment with control siRNA and the second lane represents treatment with a pool of siRNA against MBNL1. (C) Quantification of the MBNL1 immunoblot (in triplicate) plotted against log[dox]. (D) Schematic showing a theoretical example of the dose-dependent relationship between log[MBNL1] and percent spliced in (psi, Ψ), where MBNL1 levels are determined by Western relative to GAPDH. Curve fitting parameters EC50, slope, Ψ_min_, and Ψ_max_ are illustrated.

**Fig 2:**
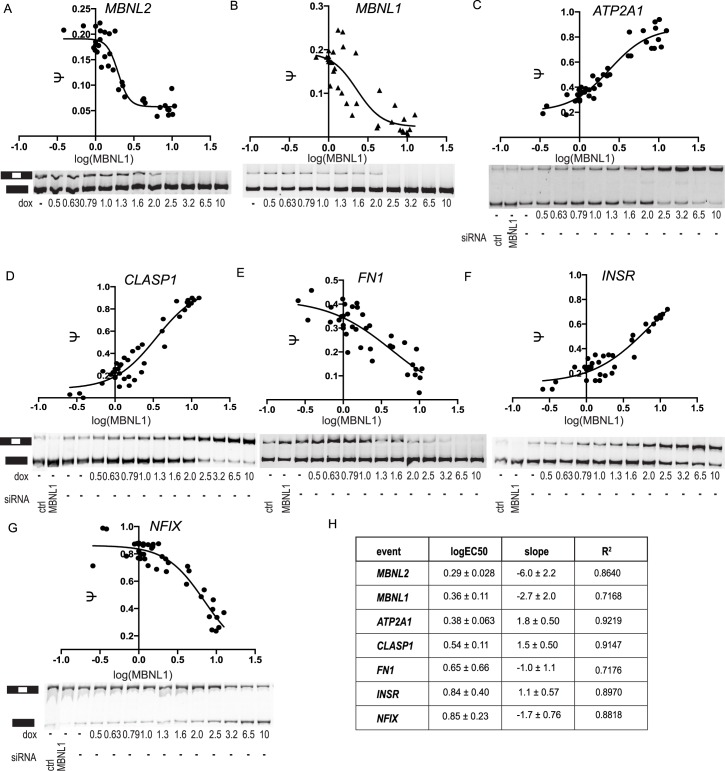
MBNL1 dose-responses. (A-G) Dose-response curves and splicing gels of individual events (A) *MBNL2* (B) *MBNL1* (C) *ATP2A1* (D) *CLASP1* (E) *FN1* (F) *INSR* (G) *NFIX*. Dox (ng/ml) was titrated to induce MBNL1 expression in HEK293 cells or treated with control siRNA or siRNA against MBNL1. siRNA treatment is not shown for events with transcripts that would be targeted by knock-down (MBNL1/2). RNA was isolated from the cells, RT-PCR was performed and DNA products were resolved on a native gel and isoform ratios quantified. Ψ values were plotted against the log(HA-MBNL1) treatment and fitted to a four-parameter dose-response curve. Splicing assays and western blots for MBNL1 protein quantification were performed in triplicate. (H) Curve fitting parameters are shown in the table.

**Fig 3:**
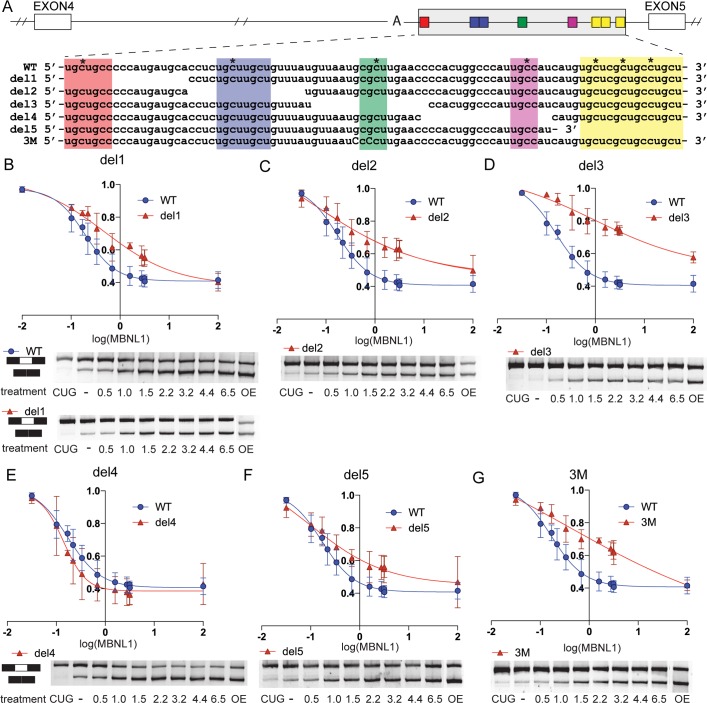
The composition of MBNL1 binding sites mediates dose-dependency of MBNL1 exon 5. (A) Mini-gene reporter sequence (exons 3-4-5 and intervening introns) of MBNL1 pre-mRNA showing the 3' region of the intronic, ultraconserved sequence between exon 4 and the regulated, or alternative, exon 5. YGCYs are indicated by colored boxes. The previously mapped distant branch point adenosine is indicated with an A [24]. Asterisks indicate C-T mutations from C2C12 cells (CLIP data, [29] (B-G) Mini-gene reporter dose-response curves were plotted (Ψ verses the log([HA-MBNL1]), determined by western blot) for (B) del1 (C) del2 (D) del3 (E) del4 (F) del5 (G) 3M in triplicate. The WT (blue) dose-response curve is included with each deletion mutant (red) for comparison. Quantification of the upper band (exons 4-5-6) and lower band (exons 4–6) were used to determine Ψ. A transiently transfected HA-MBNL1 plasmid was used to achieve the highest MBNL1 dose in this experiment and CTG_960_ transient transfection was used to achieve the lowest levels of functional MBNL proteins through sequestration. Representative splicing gels are shown.

A tetracycline-inducible Flp-In T-REx system (Invitrogen) was utilized to express HA-tagged MBNL1 in HEK293 cells (Fig 1A), to allow precise control of HA-MBNL1 concentrations as a function of doxycycline (dox) (0.5 ng/ml to 10 ng/ml). HA-MBNL1 expression covered a broad range (Fig 1B). Consistent with the low detection of MBNL1 protein in untreated HEK293 cells, low MBNL1 protein levels have been described in the literature in the kidney compared to skeletal muscle and heart in human [8,16]. Knock down of MBNL1 and sequestration of MBNL1/2/3 proteins or expression of 960 CUG interrupted repeats resulted in only minimal or modest changes in the percentage of splicing inclusion ∆Ψ for the splicing events tested (Figs 2 and 3), also consistent with low MBNL protein expression in HEK293 cells and that MBNL1 is the predominant paralog. Quantification of transcripts from HEK293 RNA seq data confirmed that MBNL1 transcripts were expressed at higher levels than MBNL2/3 (S1 Fig). This system allowed us to generate a twenty-fold dynamic range of MBNL1 protein concentration and activity in cells.

Expression of MBNL1 was achieved at lower concentrations of doxycycline (dox) than typically used for tet-on experiments (> 5 ng/ml). A sigmoidal-shaped MBNL1 concentration curve was observed when steady-state MBNL1 levels were plotted against the log of the dox concentration (Fig 1C). Maximal MBNL1 protein accumulation was approximately ten-fold higher than endogenous levels (Fig 1B) and the concentration was reduced two-fold using siRNAs to knock down the expression of endogenous MBNL1 to achieve the minimal concentrations (Fig 1B).

A schematic representation of a typical dose-response curve used to study a hypothetical MBNL1 regulated cassette exon, where MBNL1 promotes skipping, is shown in Fig 1D. The percent spliced in value (Ψ) is plotted as a function of the log of the MBNL1 dose. This schematic curve was fitted to a four-parameter dose-response equation so that parameters that relate to biological phenomena, i.e. concentration or EC50 and steepness of response, could be used to describe the dose-response data (Fig 1D). The slope of each curve provides information about the responsiveness of an event over the applied MBNL1 concentration range while the EC50 describes the relative amount of protein required for splicing activity.

### MBNL1 target exons exhibit dose-dependency, and differ in the amount of MBNL1 required for regulation

HA-MBNL1 levels were controlled with the dox-inducible system to generate dose-response curves of seven MBNL1 regulated cassette exons within genes that are expressed in HEK293 cells (Fig 2). Six of the selected events were previously validated as MBNL regulated exons and are well characterized in the DM1 field: *MBNL1* exon 5, *MBNL2* exon 5, *ATP2A1* exon 22, *FN1* exon 25, *INSR* exon 10, and *NFIX* exon 7 [10,22–28]. We also studied *CLASP1* exon 19, an exon that was identified to be dysregulated in the DM1 patient data set that is described below. The *CLASP1* event, to our knowledge, has not been described previously. MBNL1 promotes exon inclusion of the *CLASP1* exon in this study likely through binding to YGCYs within the 5' region of the intron located downstream of the regulated exon; this observation is consistent with MBNL1 binding adjacent to enhanced exons [10,28,29]. A previous study reported that MBNL1 promoted skipping of an exon within the *CLASP1* gene, but this event is a different exon; the YGCY motifs for the previously reported event are upstream [23].

Comparisons of dose-responses for each event revealed differences in the manner of regulation of each exon by MBNL1 (Fig 2). Exons in *MBNL2*, *MBNL1*, and *ATP2A1* required less MBNL1 protein than the other four events, yet *NFIX* required approximately three times more MBNL1 protein than *MBNL2*. Estimated EC50 values partially correlated to previously reported affinity measurements of MBNL1 protein bound to minimal RNAs. For example, exons with lower EC50s (*ATP2A1*, *MBNL1*, and *MBNL2*) tended to have stronger binding (K_D_s of 15 nM, 11 nM, and 5.8 nM, respectively [10]); exons with higher EC50s (*INSR*, *NFIX*) tended to have weaker binding (K_D_s of 120 nM and 55 nM, respectively) [10], [11]). Absolute values of slopes ranged from 1.0–6.0, with values from 1–1.8 for *FN1*, *INSR*, *NFIX*, *CLASP1* and *ATP2A1*, suggesting lack of apparent cooperativity. Interestingly, the autoregulated *MBNL1* and *MBNL2* exons exhibited steeper slopes than all other events analyzed. We did not observe a clear relationship between the number and location of YGCYs (S2 Fig) relative to EC50 or slope values.

### Binding site composition and organization mediate the dose-response behavior of *MBNL1* exon 5

The relationship between MBNL binding site organization and dose-response behavior is likely complex and depends on multiple factors, including *trans*-factor environment and organization of other *cis*-elements. However, by studying the splicing behavior of sequence variants of a single event, we could limit the impact of these variables. We mutated *cis*-elements in the intron upstream of *MBNL1* exon 5 to evaluate how putative MBNL binding sites affect dose-response behavior (Fig 3A). This event contains clusters of YGCYs within an intronic splicing silencer (ISS) that directly precedes regulated exon 5 [24,30] (Fig 3A). Previous work showed MBNL1 binding to these YGCYs in mouse C2C12 myoblasts [29] (Fig 3A). The ISS is considered to be “ultraconserved”, with 100% sequence identity between human and mouse [31]. Previously, we showed that individual regions containing YGCYs were unnecessary to achieve maximal splicing activity when eGFP-MBNL1 was overexpressed from a plasmid [24].

We asked whether altering the ultraconserved ISS YGCY organization altered MBNL1 dose-dependent splicing activity. Deletion of regions within the ISS (del1-5) altered YGCY organization and spacing of splicing signals, including a distant branch-site and the 3’ splice site (Fig 3A). Dox was titrated to induce MBNL1 expression, the del mutant pre-mRNA splicing reporters were transiently transfected, and the percentage of spliced transcripts including exon 5, or Ψ, was quantified and fit to curves (Fig 3 and Table 1). Maximum Ψ values were similar to WT for all del mutants (CUG condition, Fig 3), indicating that the mutations did not affect baseline levels of splicing. Minimum Ψ values, or those at the highest MBNL1 dose, were similar to WT for all deletion mutants except del 3, similar to previous observations using high MBNL1 concentrations [24]. However, the shapes of the dose-dependent curves were distinct across the MBNL1 concentration range. The central deletion mutant, del3, had the largest effect on the dose-response. This 18 nucleotide deletion resulted in removal of a single YGCY in the center of the ISS, and led to both a shallower slope and a greater EC50 relative to WT. To rule out additional explanations for why del3 exhibited a distinct dose-response, another minigene reporter, 3M, was created in which the YGCY (UGCGCU) motif within the del3 region was mutated to a sequence that MBNL does not bind, YCCY (UCCCCU) [32]. The dose-response for 3M was similar to that of del3.

**Table 1:** Curve fitting parameters of *MBNL1* exon 5 deletion mutant mini-genes. Parameters for EC50, slope, and R^2^ for the *MBNL1* deletion mutants were derived from curve fitting using a four-parameter dose-response curve.

*mbnl1*	EC50 (rel MBNL1)	slope	R^2^	min	max
construct	[95% CI]				
WT	0.19 [0.14 – 0.26]	-1.8 ± 0.28	0.9244	0.41 ± 0.018	0.98 ± 0.036
del1	0.48 [0.21 – 1.1]	-0.60 ± 0.14	0.9167	0.38 ± 0.047	1.0 ± 0.065
del2	1.2 [0.04 – 2.8]	-0.33 ± 0.26	0.8163	0.26 ± 0.32	1.1 ± 0.25
del3	1.6 [0.59 – 4.1]	-0.56 ± 0.18	0.8500	0.54 ± 0.058	1.0 ± 0.046
del4	0.15 [0.10 – 0.22]	-2.0 ± 0.67	0.8075	0.39 ± 0.026	0.96 ± 0.058
del5	0.29 [0.07 – 1.0]	-0.67 ± 0.27	0.7983	0.49 ± 0.049	0.95 ± 0.066
3M	1.7 [0.54 – 5.3]	-0.45 ± 0.16	0.9126	0.32 ± 0.10	1.0 ± 0.081

In contrast, del4, a deletion mutant lacking the YGCY 3' of del3, exhibited no significant changes in dose-dependency parameters. Another mutant, 4M, in which the del4 YGCY was mutated, also exhibited dose-dependent behavior similar to that of WT (S3A Fig). These results indicate that the *cis*-element organization of this region, in particular the sequence around the central YGCY, may mediate specific dose-response characteristics. Most alterations to binding motifs in this sequence space led to changes in dose-dependent behavior, including reduced slope and increased EC50, potentially through reducing cooperativity or changing RNA structure.

### MBNL concentration can be inferred using Ψ from multiple splicing events

In the HEK293 system, we observed that Ψ of each splicing event exhibits a characteristic sigmoid shape with respect to MBNL1 concentration. This well-controlled system allowed us to derive these relationships, and directly measure functional MBNL1 levels by Western blot. However, a major goal in the DM field is to estimate the functional, non-sequestered concentration of MBNL in tissue of DM patients. This metric is impossible to obtain from tissue using current technologies, as free versus sequestered pools of MBNL are dynamic. However, the dose-dependent curves we characterized suggested that Ψ could be used to infer the concentration of functional MBNL in cells. While we observed that *cis*-element organization plays an important role in dictating the shape of each dose-response curve, the *trans*-factor environment also likely plays a role, and therefore dose curve parameters will vary across tissues.

**Fig 4:**
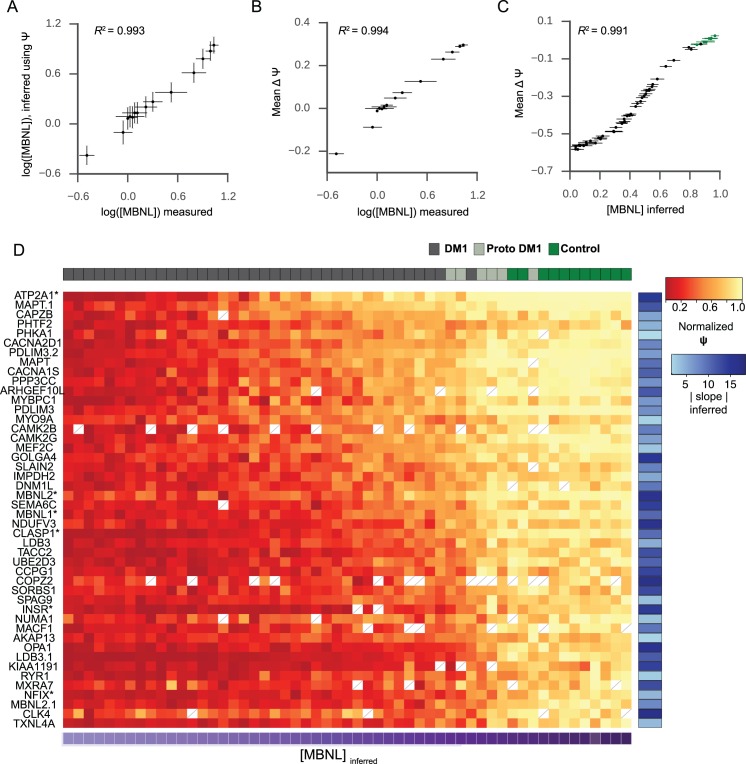
Inferring MBNL concentration using Ψ in HEK293 and DM1 tibialis anterior. (A) [MBNL] inferred using Bayesian estimation highly correlates to measurements of MBNL protein relative to GAPDH as assessed by Western blot in the HEK293 system. (B) Total splicing dysregulation, mean ΔΨ, correlates strongly with measurements of MBNL protein relative to GAPDH in HEK293. (C) Bayesian estimation was used to infer [MBNL] in 55 tibialis anterior biopsies; [MBNL]_inferred_ values also strongly correlate with total splicing dysregulation, mean ΔΨ, in tibialis. Non-DM1 individuals are shown in green. (D) Heat map of normalized Ψ (each event was set from 0 to 1, to aid visualization) for forty-six splicing events in forty-four DM1 patients and eleven healthy controls. Similar, non-normalized data is shown in S4 Fig. Ψ_min_, Ψ_max_, EC50, and slope values were inferred simultaneously with [MBNL] using Bayesian estimation (S3 Table). Samples are sorted by [MBNL]_inferred_ across the horizontal axis, and splicing events are sorted by EC50 along the vertical axis. The |slope| for each event is indicated on the right-hand vertical axis. White boxes with slashes denote samples with insufficient read coverage to infer Ψ. Events studied in HEK293 are marked with an asterisk.

First, we assessed whether it was possible to infer MBNL concentration, given a set of Ψ values measured across a range of MBNL doses. We framed the task of estimating [MBNL] and all sigmoid curve parameters, Ψ_min_, Ψ_max_, EC50, and slope, as a Bayesian estimation problem. Since we can compute the likelihood of observing Ψ for all seven splicing events from HEK293, given any set of values for [MBNL], Ψ_min_, Ψ_max_, EC50, and slope for each splicing event, Bayes’ Rule allows us to invert the problem to obtain the posterior probability distribution of each of those parameters, including the underlying MBNL concentration. Indeed, when estimated using this approach, inferred [MBNL] correlated extremely well (*R*^2^ = 0.993) with measured MBNL levels relative to GAPDH, as assessed by Western blot (Fig 4A). Interestingly, a computationally simpler approach using the average splicing dysregulation across all splicing reporters, mean Δ Ψ, also correlates extremely well with MBNL levels as assessed by Western blot (*R*^2^ = 0.994, Fig 4B).

### Estimation of functional MBNL concentration in DM1 tibialis biopsies

After using the dose-dependent relationship of Ψ to [MBNL] in the HEK293 system to develop a method to estimate [MBNL] using Ψ alone, we sought to apply this approach to measuring functional MBNL concentration in the tibialis anterior skeletal muscle, a tissue preferentially affected in DM1. Cognizant that the behavior of each dosing curve would differ in tibialis as compared to HEK293, we separately characterized dosing curves in tibialis, by analyzing RNA-seq data from 44 DM1 patients (including 5 patients with proto-mutations, or <100 CTG repeats as assessed by peripheral blood) and 11 healthy controls (sample cohort described in [17]). MISO was used to derive transcriptome-wide estimates for Ψ values of alternative cassette exons [33]; we focused on 46 cassette exon splicing events exhibiting significant differential splicing regulation in at least 25% of DM1 samples (*MISO Bayes Factor* ≥ 5 when comparing DM1 to non-DM1, and |Δ*ψ*| > 0.2) (S1 and S2 Tables). MISO Ψ estimates were in agreement with values obtained from RT-PCR [17].

While in the context of DM1 many factors potentially influence ΔΨ, the panel of events identified in this study is consistent with a predominant contribution from MBNL sequestration. YGCY motifs were enriched upstream of the regulated exon for events that are more included in patients (p = 0.001) while YGCY motifs were enriched downstream of the regulated exon for events that are more excluded in the patients (p = 0.006), consistent with previously described MBNL1 binding site maps and observations connecting MBNL1 putative motif location and association with either inclusion or skipping of the alternative exon [10,28,29]. Additionally, previous studies indicate that MBNL depletion accounts for the majority of splicing events observed in the HSA^*LR*^ mouse model of DM1 [28].

Six out of seven splicing events studied in the HEK293 system were also observed to be dysregulated in DM1 tissue; *FN1* was excluded because it lacked sufficient RNA-seq coverage to accurately infer splicing. To characterize dosing curves in tibialis, we applied our Bayesian inference framework, which infers Ψ_min_, Ψ_max_, EC50, and slope for each splicing event in tibialis, as well as functional MBNL concentrations for each individual. As expected due to differences in *trans*-factor environment and expression level, among other variables, dose-dependent curves were observed to be similar, but not identical between HEK293 and tibialis anterior (S5 Fig). Interestingly, while curve shapes were similar across both cell types, those in tibialis generally exhibited a broader dynamic range in Ψ, as well as steeper slopes, suggesting that the *trans*-factor differences in tibialis result in an enhanced dependency of Ψ on MBNL concentration.

Similarly to the HEK293 system, we observed that the intracellular concentration of functional MBNL ([MBNL]_inferred_) correlated well with mean splicing dysregulation (Fig 4C); this is simply a reflection of the fact that the Bayesian Inference approach aggregates Ψ from multiple events to generate its estimates. Ordering individuals by relative [MBNL]_inferred_ resulted in non-DM1 individuals grouping together with individuals carrying DM1 proto-mutations, consistent with limited MBNL sequestration occurring in this subset of DM1 patients (Fig 4D). Some events showed dysregulation in almost all patients (86% of patients exhibit dysregulation for *INSR*), and others showing dysregulation in a smaller subset of patients (48% of patients exhibit dysregulation for *ATP2A1*), consistent with some events exhibiting higher EC50 values than others (Fig 4D and S1 Table). Ψ for all samples and all splicing events, along with estimated sigmoid curves, are shown in S6 Fig. Although reported repeat lengths [17,34–36] for a subset of fifteen TA muscle samples did not correlate to [MBNL]_inferred_ values (*R*^2^ = 0.0841) (S7A Fig) or muscle weakness (17), it is well established that repeat lengths can differ from cell to cell of the same individual, and that long repeats are extremely difficult to size accurately. However, ankle dorsiflexion (ADF) strength measurements were moderately correlated with [MBNL]_inferred_ (*R*^2^ = 0.358) (S7B Fig).

### Accurate inference of MBNL concentration is splicing event and disease severity dependent

**Fig 5:**
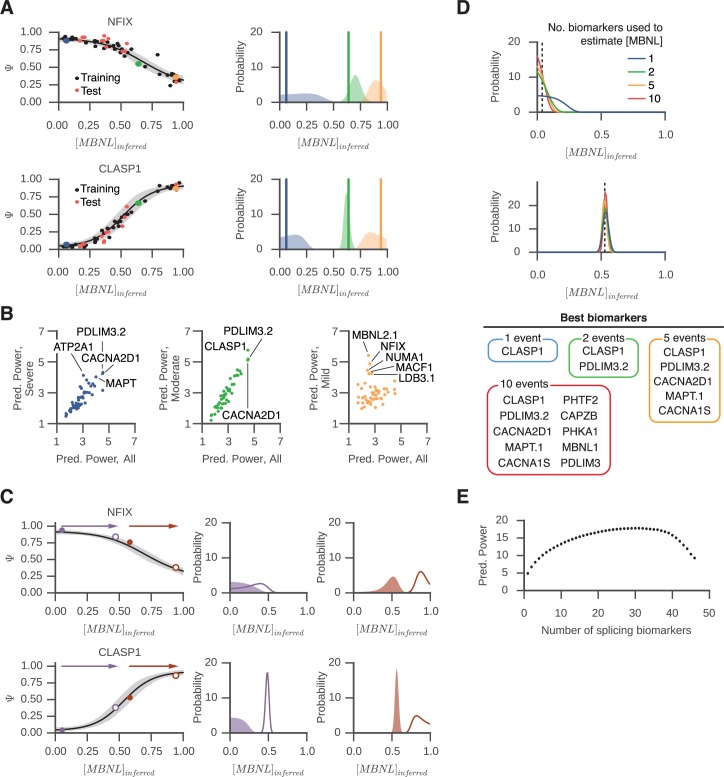
Splicing events as biomarkers to measure functional MBNL concentration in DM1 muscle and response to therapeutics. (A) Ψ_min_, Ψ_max_, EC50, and slope, as well as [MBNL], were estimated for each splicing event and each sample, using 70% of the tibialis biopsies using a Bayesian inference framework. Sigmoid curves with 95% confidence intervals for *NFIX* and *CLASP1*, as estimated from 70% of the data, are shown, along with the Ψ values used to derive the curves (black points). Posterior distributions for [MBNL] were derived for 30% of the data (red points), and plotted for 3 specific samples (blue, green, and orange points). These distributions are also plotted on the right, along with the “gold standard” [MBNL] as estimated using 100% of the data (blue, green, and orange vertical lines). (B) The mean predictive power of each splicing event to predict “true” [MBNL] was calculated across 120 random subsets of training and test sets, where 70% of samples were used for training, and 30% for testing. Predictive power was defined as the posterior probability estimate at “true” [MBNL] assessed using all the data. The mean predictive power was computed separately across three patient subgroups–severe ([MBNL] < 0.33), moderate (0.33 < [MBNL] < 0.66), and mild ([MBNL] > 0.66) DM1, as well as across the entire patient cohort. Mean predictive power for each patient subgroup was plotted versus mean predictive power across the entire cohort, or all patients; splicing events that perform better in specific subgroups relative to the entire cohort are labeled. (C) The ability to estimate changes in [MBNL] depends on the splicing event used to infer [MBNL], as well as the disease severity at which treatment is initiated. Hypothetical changes in [MBNL] of +0.4 and +0.3 during a therapeutic trial are illustrated as purple and brown filled and outlined points, respectively in left panels. The posterior probability estimates of [MBNL] are illustrated in right panels. (D) A greater number of biomarkers can improve predictive power of estimates of [MBNL]. The posterior probability distribution for [MBNL] is shown when using 1, 2, 5, or 10 biomarkers for severely affected tibialis (top panel) or moderately affected tibialis (bottom panel). The value of [MBNL] obtained when using 100% of samples is shown as a dotted line; the posterior probability at this value of [MBNL] increases as the number of biomarkers increases. (E) Mean predictive power increases when using more biomarkers, up to a point. The best possible combination of biomarkers was chosen for each cross-validation trial, and predictive power was averaged across 120 cross-validation trials.

We sought to assess the suitability of each splicing event as a potential biomarker for levels of functional, non-sequestered MBNL, a key metric likely correlated to clinical outcomes in DM1. To simulate a hypothetical future clinical trial scenario in which we have estimated Ψ_min_, Ψ_max_, EC50, and slope using one cohort of DM1 patients, and would like to estimate [MBNL] for a new cohort of patients, we divided our samples into two groups to perform traditional cross-validation. We used 70% of the individuals to estimate Ψ_min_, Ψ_max_, EC50, and slope for every splicing event (training); these trained parameters could be used to plot sigmoid curves for each event (*NFIX* and *CLASP1* shown in Fig 5A). We then assessed how well we could predict [MBNL] for the remaining 30% of samples (testing), by framing the question as another Bayesian inference problem. Here, we obtained the posterior probability distribution for [MBNL] by computing *p*([*MBNL*] | Ψ) *p*(Ψ | [*MBNL*]).

We then used the trained parameters to estimate a posterior probability distribution for [MBNL], framing the question as another Bayesian inference problem. Posterior distributions for [MBNL], obtained using measurements of Ψ for *NFIX* or *CLASP1* are displayed in blue, green, and orange shading, for Ψ values observed in 3 distinct biopsy samples (Fig 5A, right panel). The “gold standard” estimates for [MBNL], previously calculated using 100% of the data, are displayed in blue, green and orange vertical lines. Apparent from these estimates is that the precision with which [MBNL] can be predicted is dependent on the shape of the sigmoid curve. For example, a precise value for [MBNL] in a severely affected individual (based on ADF measurements) is more difficult to obtain using *NFIX* as compared to *CLASP1*, because *NFIX* offers little discriminatory power between 0 and 0.5 [MBNL] units. In contrast, the predictive power of *NFIX* is slightly better than that of *CLASP1* for mildly affected patients, as the sigmoid curve for *NFIX* changes more steeply than *CLASP1* at the high end of [MBNL]. *CLASP1* is an excellent predictor in the moderate range (green); the posterior probability estimate at the “true” [MBNL] value is close to 7 (Fig 5A).

These examples illustrate the principle that some splicing events are better than others for predicting [MBNL], and that their predictive power is dependent on disease severity. To quantitate the performance of each splicing event to estimate [MBNL], we calculated the mean predictive power of each splicing event to estimate “true” [MBNL] across 120 randomly sampled 70%/30% training/testing cohort divisions, where predictive power is defined as the posterior probability estimate at “true” [MBNL]. We calculated the mean predictive power for each event across several patient subgroups–severe ([MBNL] < 0.33), moderate (0.33 < [MBNL] < 0.66), and mild ([MBNL] > 0.66) DM1, as well as across the entire patient cohort. Splicing events best suited to predict [MBNL] in mild DM1 are distinct from those best suited to predict moderate or severe DM1 (Fig 5B). Interestingly, splicing events best suited to predict [MBNL] in moderate DM1 are also those best suited to predict [MBNL] across the entire spectrum (*CLASP1*, *PDLIM3*.*2*, *CACNA2D1*). Analyses of sigmoid curves for the best performers (S4 Fig) indicated that the best predictors exhibit non-zero slopes within the range of [MBNL] being predicted.

### Measurement of therapeutic rescue requires proper biomarker selection, and usage of multiple biomarkers improves measurement accuracy

It is well established that the goal of many therapeutic efforts for DM is to increase the free, functional concentration of MBNL in cells and tissues; therefore, it is critical to be able to measure changes to this metric before and after a clinical study/trial. A hypothetical change in [MBNL] may or may not lead to changes in Ψ for any given splicing event; the change in Ψ is dependent on the shape of the sigmoid curve, as well as the starting point and ending point within the curve (Fig 5C). For example, a positive change of 0.4 [MBNL] units for a severely affected DM1 patient will result in changes to Ψ for *CLASP1* but not *NFIX* (Fig 5C, left panels, purple points and arrows). Posterior estimates for pre- and post-treatment [MBNL] using sigmoid curve parameters are poorly separated for *NFIX*, yet well separated for *CLASP1* (Fig 5C, purple shading). In contrast, a positive change of 0.3 [MBNL] units for a moderately affected DM1 patient results in well separated pre- and post-treatment [MBNL] estimates for both *NFIX* and *CLASP1* (Fig 5C, brown data points).

Thus far, our analysis has made use of only one biomarker at a time to estimate [MBNL]. We tested the ability to improve estimates by incorporating multiple biomarkers; we essentially computed the joint probability distribution of [MBNL] as estimated by each separate biomarker. We identified biomarker combinations that would maximize predictive power across all disease severities, again using 70%/30% cross-validation training/test sets. For each cross-validation trial, we first identified the single best biomarker with maximum predictive value for [MBNL]; we then identified the next best biomarker that minimized errors in concert with the best biomarker, then the third in concert with the first two, and so on. We performed this analysis across all 120 cross-validation trials, and recorded the biomarker sets with highest mean predictive power across all trials. Shown in Fig 5D are posterior estimates of [MBNL] for a severely affected biopsy and moderately affected biopsy, using the best 1, 2, 5, or 10 biomarkers (Fig 5D). As expected, the probability distribution becomes sharper and more centered on the “true” [MBNL]. Adding more biomarkers can improve the estimation up to ~30 markers (Fig 5E), after which further marker addition decreases performance. The decrease in performance is likely due to use of Ψ values from splicing events whose inclusion is not as tightly linked to [MBNL].

## Discussion

### Splicing events exhibit distinct responses to MBNL concentration

**Fig 6:**
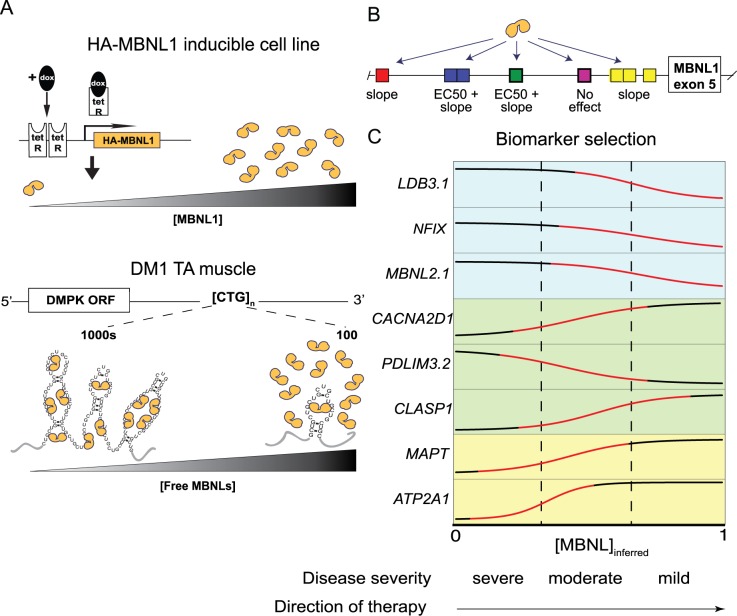
Summary. (A) In our HEK293 inducible model, free [MBNL1] can be titrated by administration of doxycycline (top panel). In myotonic dystrophy, free [MBNL] proteins are titrated by sequestration to toxic CUG RNA (bottom panel). (B) Deletion of YGCY sites within the ultraconserved intron upstream of MBNL1 exon 5 influences dose-dependent curve parameters EC50, slope, both, or neither. (C) Biomarkers are most informative in the regions with a non-zero slope (red parts of curve). The best biomarkers also tend to have a broad dynamic range (Ψ_max_- Ψ_min_).

We studied the behavior of 7 splicing targets in a cell line we developed, in which MBNL1 expression could be titrated across a 20-fold dynamic range (Fig 6A). Each splicing event exhibited a unique, sigmoidal dose-response curve, with distinct EC50 and slope parameters. The steepness, or slope, of Ψ relative to [MBNL1] varied across splicing events, likely due to cooperative binding of MBNL1 and/or other proteins to pre-mRNA. MBNL1 is known to regulate the splicing of other splicing factors such as PTB1 and hnRNPA1 and the stability of transcripts encoding splicing factors [29], so it is possible that secondary effects play a role in producing the observed sigmoid curves.

One might hypothesize that multiple binding sites are more likely to function in a cooperative fashion and that high affinity binding would lead to a lower EC50, but we did not observe a simple relationship between the number and location of MBNL binding sites and EC50 or slope. The location of MBNL bound *cis*-elements is similar when comparing MBNL1 exon 5 and MBNL2 exon 5, and they exhibited similar dose-dependent responses (Fig 2 and S2 Fig). The relationship between MBNL binding site number, location and affinity almost certainly play an important role in determining MBNL1 dose-responses, but it is also likely that other splicing factors, RNA structure and gene expression levels can modulate these curves. Other factors that regulate splicing in DM1 TA muscle, including CELF1 proteins, will also affect the curve parameters_._ CELF1 antagonistically regulates some MBNL regulated events including *INSR* [37–39].

### The relationship between MBNL binding site organization and dose-dependent behavior

We studied the role of specific binding sites upstream of *MBNL1* exon 5 in mediating its MBNL dependent dose-response (Fig 3). Inclusion of *MBNL* exon 5 controls localization of the protein, and appears to be tightly regulated by MBNL and other proteins [29,40,41]. This region is an “ultra-conserved region” [31], and previous work has shown that in mouse myoblasts, MBNL1 protein interacts with the region mutated in these experiments [29]. Some YGCY motifs were dispensable for splicing when MBNL1 was high [24], but at low concentrations, some motifs were more critical than others.

The site that most dramatically altered dose-dependent behavior was a single, central YGCY. Affinity and cooperativity parameters were reduced for the del2/3 regions while del1/5 only had reduced cooperativity (Fig 6B). MBNL1 may bind to central sites first causing the surrounding sites to become more accessible, consistent with the footprinting data of this RNA (Fig 6B and S3B Fig). Structure probing indicated that the central YGCYs were single stranded in vitro ([24] summarized in S3B Fig) and that the RNA was generally not structured. Notably, previous studies indicate that MBNL1 binds to regions of short single stranded RNAs through the Watson-Crick face of short unstructured RNAs [10,32,42]. Alternatively, differences in structure between the del mutants may have contributed to the observed differences in EC50 and slopes observed (S3B Fig) (mfold server, [43]). For example, the open structures for WT and del4 may facilitate a cooperative dose-response and increased sensitivity to MBNL1 concentrations, while the alternative structure may be a weaker substrate for MBNL1 (S3B Fig). Indeed, recent data suggests a model where MBNL1 interacts with the toxic CUG RNA when it is destabilized by U-U mismatches, allowing MBNL1 to access locally unfolded GC sites [44,45].

### Alternative splicing event biomarkers

A key goal in the DM field is to accurately assess the remaining functional levels of MBNL proteins in human tissue. Using the cell-based system, we developed and validated a computational method to estimate MBNL1 concentration, as well as splicing curve parameters, using Ψ alone. The strong correlation between estimated and measured MBNL1 protein levels in the cell-based system motivated us to take a similar approach in human tibialis biopsies from DM1 affected and non-affected individuals, where free MBNL proteins vary between individuals (Fig 6A). We estimated tibialis-specific splicing curve parameters, which differed from those observed in the cell-based system, and also estimated the levels of functional MBNL protein remaining in tissue. As MBNL proteins regulate the splicing of many pre-mRNA substrates, a major unresolved question is which splicing events are most informative about disease status, and which splicing events in combination are best suited to measure therapeutic rescue in a clinical trial. It is well established that the remaining functional level of MBNL protein in tissue is informative about disease status, and here, we demonstrate that we can estimate this value using Ψ alone, provided we first build a model using Ψ from a number of splicing events across a spectrum of disease severities.

We showed that the range of [MBNL] across which Ψ varies is different across events, and that this characteristic determines which subset of patients (mild, moderate or severe) for which that biomarker exhibits the greatest predictive power (Fig 6C). This has implications for being able to accurately place patient samples along the continuum of disease severities observed in DM, and for being able to accurately measure the extent of therapeutic rescue in a clinical trial. Furthermore, splicing biomarkers whose Ψ values are invariant across a particular [MBNL] range do not provide as much information as biomarkers whose Ψ values change dramatically across that same range. Finally, we showed that using multiple biomarkers in combination to predict [MBNL] holds more predictive power than using single biomarkers alone. The best biomarkers significantly overlap with those previously described [17]; we found the top set of five biomarkers for tibialis to be *CLASP1*, *PDLIM3*.*2*, *CACNA2D1*, *MAPT*.*1* and *CACNA1S* (Fig 5D and S3 Table). Interestingly, *CLASP1* is a novel event identified in our RNA-seq analysis; it is the top biomarker among all tested, and exhibits a broad range of splicing regulation across [MBNL] in both the cell based system and in tibialis. (Fig 2 and S6 Fig).

### Dose-dependent behavior differs in different cellular contexts

The relationship between MBNL1 levels and Ψ was previously investigated in myoblasts and mouse muscle [20]. Ψ for five splicing targets was measured at five concentrations of MBNL1 in myoblasts, achieved by different doses of siRNA; Ψ for similar events was measured at 0%, 50% and 100% MBNL1 level in mouse muscle, using normal, heterozygote, and homozygote MBNL1 knockouts. The relationship of Ψ to [MBNL1] was slightly different in myoblasts versus muscle, depending on the specific exon, suggesting that a complex mixture of cis- and trans-factors mediates dose-dependent behavior, with differing stoichiometry in different cellular contexts. Here, we have separately measured Ψ_min_, Ψ_max_, EC50, and slope values for each splicing event in both HEK293 cells and human tibialis, and also observed that these parameters differ between HEK293 cells and human tibialis. These observations suggest that proper selection of splicing biomarkers for a given cell type requires characterization of biomarkers in that tissue, or a basic understanding of how Ψ is modulated by the interaction of multiple *trans*-factors with pre-mRNA *cis*-elements.

## Materials and Methods

### HA-MBNL1 expression stable cell line production

The full length MBNL1 (isoform 41) with an N-terminal HA tag was cloned into the supplied vector (pcDNA5) and transfected into the HEK293 T-REx FLP cell line (Life Technologies) to create the inducible line following the manufacturer's protocol.

### Western blotting

HEK cell pellets were lysed in (RIPA) buffer (50 mM Tris, pH 7.4, 150 mM NaCl, 1% NP-40, 0.25% sodium deoxycholate, 0.1% SDS, 1 mM phenylmethylsul- fonyl fluoride [PMSF]) supplemented with 1x protease inhibitor cocktail (SigmaFAST; Sigma) by light agitation for 20 min via vortex, and the concentration of protein was normalized using bicinchoninic acid (BCA) reagent (Pierce) prior to resolution on 10% SDS-PAGE gels. MBNL1 proteins were probed with antibody (MB1a (4A8)) [46] probed at 1:2000 and 1:15,000 goat anti-mouse secondary IRDye 800CW (Licor). GAPDH loading control was probed (1:1000) ((14C10) Rabbit mAb #2118, Cell Signaling) followed by goat anti rabbit IRDye 680 RD (Licor). Fluorescence was measured using a Licor Odyssey Fc instrument.

### Splicing reporters and mini-gene mutagenesis

The wild type MBNL1 mini-gene was made by amplifying regions of the MBNL1 gene containing 51 nucleotides from the 3'-end of intron 3, exon 4, intron 4, exon 5, intron 5, exon 6, and 33 nucleotides of the 5' -end of intron 6 from HeLa genomic DNA using PCR primers with unique restriction sites. *Mbnl1* deletion constructs are described previously [24]. Site directed mutagenesis of the WT and del mutants constructs was performed to create the mutations 3M and 4M using the Phusion Site-Directed Mutagenesis Kit following the manufacturer’s protocol (Thermo Scientific). The following primers were used for mutagenesis: 3M: Forward: 5’- GTTTATGTTAATCCCCTTGAACCCCAC -3’, Reverse: 5’-AGCAAGCAGAGGTGCATC ATG-3’, 4M: Forward: 5’- CTGGCCCATTCCCATCATGT -3’ Reverse: 5'-TGGGGTTCAAGC GCATTAACAT-3'.

### Cell culture and transfection

HEK293 cells with inducible MBNL1 were routinely cultured as a monolayer in Dulbecco’s modified Eagle’s medium (DMEM)-GlutaMax (Invitrogen) supplemented with 10% fetal bovine serum (Gibco) at 37°C under 5% CO2. Cells were plated in twenty-four-well plates at a density of 1.5 x 10^5^ cells/well. Fresh doxycycline (Sigma) or tetracycline (Sigma) was prepared at 1 mg/ml, diluted, and added to the cells at the appropriate concentrations. A pool of three siRNA duplexes (CACUGGAAGUAUGUAGAGAdTdT, GGACAAAUGUGCUUGGUUUUUU, and GAGAGAAACCUGUAUAAUAUU) were transfected into the cells 24 hours prior to harvesting cells using TransIT-siQUEST transfection reagent as per the manufacturer's protocol. Prior to transfection, cells were plated in twenty-four-well plates at a density of 1.5 x 10^5^ cells/well. Cells were transfected 24 h later at approximately 80% confluence. Plasmid (500 ng/well) was transfected into each well with 1.5 ul of TransiT 293 (Mirus) following the manufacturer’s protocol. 250 ng of either empty vector (pcDNA3.1+) or DMPK-CTG_960_ was co-transfected into a single well with 250 ng of mini-gene reporter for all splicing assays. For DMPK-CUG_960_ and HA-MBNL (pcDNA3.1+) each reporter was reduced so that the total DNA transfected remained constant and the ratio of DNA to transfection reagent remained constant. Fresh doxycycline (Sigma) or tetracycline (Sigma) was prepared at 1 mg/ml, diluted, and added to the cells at the appropriate concentrations four hours post transfection. The DMPK-CUG_960_ plasmid was obtained from the laboratory of Thomas Cooper.

### Splicing activity assays

Cells were lysed in the plate and RNA isolated using an RNeasy kit (Qiagen) twenty hours after induction. Isolated RNA (200 ng) was reverse transcribed using Superscript II (Invitrogen), according to the manufacturer’s protocol. The RNA for all endogenous splicing event conditions were internally controlled by recovering RNA from the same plated well of cells. Endogenous genes were reverse transcribed using random hexamers except for MBNL1, which a gene specific primer was designed to anneal to endogenous transcripts but not the HA-MBNL1 transgene transcripts:

5’-CTGAGGAACTTTTGTGTGTGTTGCTTGACG-3’. All reverse transcription reactions were subjected to PCR amplification in a 20 ul reaction mixture using flanking exon-specific primers. The number of amplification cycles was determined to be within the linear range for all primers used. Endogenous gene PCR primers (hg19 coordinates are the same as in S2 Table) are as follows: ATP2A1 primers are described in Purcell et al [11],

INSR: F: 5’-CCTGTCCAAAGACAGACTCTCAGATCCTG-3’

R: 5’-GTCGAGGAAGTGTTGGGGAAAGC-3’, CLASP1:

F: 5’-CAAAGTCTCCTCATCTTCGGGCACG-3’

R: 5’-GCTGGGACTGTGAAACCACTTTAGC-3’, MBNL1: F 5-AGGGAGATGCTCTCGGGAAAAGTG-3’, R 5’-GTTGGCTAGAGCCTGTTGGTATTGGAAAATAC-3’, MBNL2 (from [17]): F 5’-ACAAGTGACAACACCGTAACCG-3’, R 5’-TTTGGTAAAGGATGAAGAGCACC-3’, NFIX: F 5’-GATGGAGAGCCCTGTTGATGACG-3’ R 5’-GTGGTGGTGGTAGCGGATGGTC-3’ and FN1 (exon hg19 coordinates chr2:216257654–216257926) F 5'- AGAATTACCACAACCCCTACAAACGG -3' R 5'- TGCATTGTCTGAAGGAGAAATTGACAACTC -3'.

PCR products were resolved by gel electrophoresis on 1.5-mm, 6% native polyacrylamide (19:1) gels run at 300 V for 90 min. Splice products were visualized and quantified using SYBR green I nucleic acid stain (Invitrogen) in combination with an AlphaImager HP system (Alpha Innotech). All reported values were obtained from at least three independent splicing experiments. All experiments with MBNL1 pre-mRNA mini-gene mutations included a WT internal control.

### RNAseq

Muscle biopsies, repeat length estimation, and patient strength measurements were conducted as described in Nakamori et al [17] and [47]. 1 ug of total RNA was prepared for Illumina strand-specific RNA-seq using standard methods. In brief, rRNA was removed using the Ribozero beads (Epibio), and remaining RNA was fragmented using fragmentation buffer (Ambion #AM8740). RNA was precipitated and converted to cDNA using Superscript III, random primers, and dUTP, and 2nd strand cDNA using DNA polymerase I and RNase H. Ends were repaired, adenylated, and ligated to Illumina paired end adapters. Ligated fragments were purified by agarose gel electrophoresis (~200 bp band was excised), treated with USER enzyme, and subjected to 14–18 cycles of PCR. A second gel was run to purify product away from primers. Libraries were sequenced in pooled sets of 4 or 6 samples on the Illumina Hiseq, using 2 x 57 bp sequencing runs.

### Identification of mis-spliced exons in DM1 patient samples

Illumina RNA-sequencing reads from the tibialis anterior muscle biopsies (44 DM1 and 11 unaffected control) were aligned against the *hg19* human reference genome with GSNAP and allowing for novel splicing [48] (http://research-pub.gene.com/gmap/). GSNAP was run with the following options set:–s [*splice sites map file*]–N 1 –A sam–o FR—pairexpect 300—pairdev 100. A splice sites map file was generated from the *hg19* gene models (GrCh37 release 75). Isoform abundances were estimated and each of the 44 DM1 samples was compared to each of the 11 control samples using MISO [33]. A custom set of *hg19* alternative event annotations were generated from the Refseq, Ensembl, and UCSC gene models using custom scripts. For this analysis we focused only on the differential inclusion and exclusion of “cassette” exons between the DM1 and control samples. The comparison data was filtered based on the criteria described below to identify a high confidence set of misregulated alternative exons events.

Filtering criteria:

1. ≥ 70% of control and DM1 samples have sufficient coverage over the event.
  - ∑ inclusion and exclusion reads ≥ 20
2. $|\bar{\Delta \psi }|\geq 0.20$
3. ≥ 25% of DM1 patients showed evidence for differential splicing compared to controls.
  - median Bayes factor ≥ 5

### Curve fitting (Figs 1–3)

For data in Figs 1–3, splicing activity curves were fitted using Graphpad Prism software using non-linear curve fitting (log(agonist) vs. response—Variable slope (four parameters)). The min and max were restricted to fall between 0 and 1, respectively.

### YCGY motif enrichment near differentially regulated exons

A student's t-test was used to compare the presence of 4-mer motifs that bind MBNL proteins (identified previously [10]), using a 20 bp sliding window within the 400 bp region upstream of cassette exons that are more excluded in controls, with respect to patients, to the same region of an identical number of randomly sampled exons (average sampled using 1000 iterations). A similar comparison was performed with motifs located 400 bp downstream of cassette exons that are more included in controls, with respect to patients.

### Bayesian inference to estimate curve parameters and [MBNL]

We modeled the problem using Bayes’ Rule as follows: $p(\phi |\vec{\psi })\propto p(\vec{\psi }|\phi )*p(\phi )$ where $\vec{\psi }$ is the observed inclusion level of an exon across multiple doses or samples, and *ϕ* describes Ψ_min_, Ψ_max_, log(EC50), slope, [MBNL], and a parameter σ. We assume that observed Ψ values are drawn from a normal distribution centered around the modeled Ψ value, with standard deviation σ. Priors for each estimated parameter were as follows: Ψ_min_ ~ Uniform(0, 1), Ψ_max_ ~ Uniform(0, 1), log(EC50) ~ Normal(μ = 0.5, σ = 1), slope ~ Normal(μ = 0, σ = 1), [MBNL] ~ Uniform(0, 1), σ ~ Uniform(0, 1). The python package PyMC3 was used to implement Bayesian inference [49]. MCMC sampling was performed using the NUTS sampler and 1000 iterations. This approach was used to infer [MBNL] for all panels in Fig 4, separately for HEK293 data and tibialis data. Estimated parameters were used to generate curves in S4 Fig.

### Cross-validation analysis, generating posterior estimates of [MBNL], and biomarker analysis

We assessed the ability of each splicing event to predict [MBNL] by performing a cross-validation analysis. We divided the tibialis samples into 2 groups, comprising 70% and 30% of samples. Ψ_min_, Ψ_max_, log(EC50), slope, [MBNL], and σ were estimated using 70% of the samples, as described above. Then, for each splicing event and each sample in the remaining 30% of samples, we calculated a posterior distribution for [MBNL]. We essentially performed Bayesian inference again, framing the problem as computing the probability of [MBNL] across all possible values of [MBNL]. That is, we computed *p*([*MBNL*]|*φ*,*ω*) ∝ *p*(*φ*|[*MBNL*],*ω*) * *p*([*MBNL*],*ω*). Here, *ω* describes Ψ_min_, Ψ_max_, log(EC50), slope and σ, a parameter describing the standard deviation of the normal distribution from which observed Ψ values are drawn from the modeled Ψ value (similar to above). In this case, however, σ is directly computed from the training data, so that events with observed Ψ values that closely match predicted Ψ are more highly favored as biomarkers. Fig 5A shows example posterior distributions of [MBNL] for points highlighted in blue, green, and orange. We defined “predictive power” as the value of the posterior distribution of [MBNL] where [MBNL] equals that estimated when using 100% of the data. To compute the mean “predictive” power for mild, moderate, or severely affected samples, we averaged predictive power for samples with [*MBNL*] > 0.66,0.33 < [*MBNL*] < 0.66, and [*MBNL*] < 0.33 (Fig 5B). To compute predictive power when using multiple splicing events as biomarkers, rather than take an exhaustive approach, which would require testing upwards of billions of combinations of splicing biomarkers (for example, to sample 46 choose 10 is ~4 billion), we took a recursive addition approach, where we identified the best biomarker, then identified a second biomarker yielding the best performance in concert with the best biomarker, then a third biomarker yielding the best performance in concert with the first 2 biomarkers, and so on (the best biomarkers are shown in Fig 5D). Predictive power was computed as the joint probability distribution of all posterior estimates of [MBNL] for chosen biomarkers.

### Schematic diagrams of splicing events

YGCY motifs were plotted to scale within the regulated exon, 200 nucleotides upstream of the regulated exon and 200 nucleotides downstream of the regulated exon.

### TPM calculations (S1 Fig)

Kallisto [50] was used to estimate transcripts per million using a kallisto transcriptome index generated from the hg19 Refseq/Locuslink coding sequences (CDS) fasta data set. Paired-end FASTQ files were used for TPM quantification and the average fragment length of the elibary was automatically esimated by Kallisto. Total TPM values were calculated by summing Refeq TPM values across all corresponding Refseq IDs for a given geneID.

## Supporting Information

S1 Fig Relative transcript levels of MBNL paralogs. MBNL1/2/3 transcripts were estimated using RNA seq from six HEK293 cell samples. Transcripts per kilobase million are reported.
(TIF)

S2 Fig YGCY motifs of MBNL1 regulated events. 200 nucleotides upstream and downstream of the regulated exon are depicted with YGCY motifs marked. Schematic element spacing is drawn to scale.
(TIF)

S3 Fig Additional mutation in *MBNL1* mini-gene reporter and predicted RNA structures of wild type and mutant RNAs. (A) 4M has similar behavior to WT. Splicing of 4M mutant compared with WT *MBNL1* mini-gene reporter in triplicate. Experimental details are the same as in Fig 3. Representative gel is shown. (B) *MBNL1* intron 4 is generally unstructured. Intron 4 mapped with previously determined structural data [24]. Single stranded regions are marked with a dashed line, nucleotides with increased cleavage in the presence of MBNL1 are marked with an E, and MBNL1 protected nucleotides are marked with a P (C) Predicted secondary structures obtained using the mfold server for *MBNL1* WT and mutant RNAs [43].
(TIF)

S4 Fig Ψ Estimates for forty-six splicing events from DM1 and control tibialis muscle. Heatmap of event Ψ estimates (un-normalized) from DM1 and control RNA-seq tibialis samples. Samples are ordered from left to right by decreasing |Δ Ψ| summed over all events.
(TIF)

S5 Fig Events differ in their dose-responses between HEK293 and TA muscle. (A) Ψ was plotted against [MBNL1] as determined by western blot in HEK293 for *NFIX* (blue), *ATP2A1* (purple), *INSR* (black), *MBNL1* (red), *MBNL2* (green), and *CLASP1* (orange) (left panel) and TA muscle Ψ was plotted against inferred [MBNL] (right panel). The x-axes are log_10_ scale. Individual points and error for each event is shown is Fig 2 and S6 Fig.
(TIF)

S6 Fig Individual splicing curves in DM1 and control tibialis muscle. Ψ estimates are plotted against the inferred MBNL1 for each splicing event. Inferred dose-response curve are shown.
(PDF)

S7 Fig Strength of Ankle Dorsiflexion (ADF) but not number of CTG repeats correlates with average dysregulation. (A) CTG repeat length was not correlated with inferred [MBNL] in the tibialis muscle samples (*R*^2^ = 0.0841). (B) The maximal isometric force of ankle dorsiflexion (ADF), measurement (expressed as the percentage of strength that would be predicted in a healthy person of same age, gender, and height) moderately correlates with inferred [MBNL] (*R*^2^ = 0.358).
(TIF)

S8 Fig YGCY motifs within events misregulated in DM1 tibialis muscle. 200 nucleotides upstream and downstream of the regulated exon are depicted with YGCY motifs marker. Schematic element spacing is drawn to scale.
(TIF)

S1 Table Events misregulated in DM1 compared to unaffected control tibialis. Splicing event coordinates from MISO from hg19, gene symbol, mean Ψ for control and DM1 samples, standard deviation, mean ΔΨ, number of samples used to calculate the mean for control and DM1, number of samples for each event with a Bayes Factor greater than 5, and % patients with dysregulated splicing for each event (ΔΨ > 50%, normalized to the maximum ΔΨ for each event) are shown in the table.
(XLSX)

S2 Table Ψ estimates for events perturbed in DM1 tibialis muscle compared to unaffected controls. Ψ estimates from MISO for genes (indicated by gene symbol and hg19 coordinates) are shown for all samples in this study. NA is used for samples that did not have sufficient coverage to obtain an estimate for that splicing event.
(XLSX)

S3 Table Curve-fitting parameters for events dysregulated in DM compared to unaffected controls. Bayesian posterior mean estimates for parameters min Ψ, max Ψ, EC50, slope, respective 5% and 95% confidence intervals, and σ are shown for each splicing event (event_parameters tab). Inferred [MBNL], 5% and 95% confidence intervals, and mean delta psi are shown for each sample (MBNL_inferred tab). Biomarker predictive power for all events (Fig 5B) and optimal combinations for choosing one to forty-six biomarkers (Fig 5D) are shown (single_biomarkers and biomarker_combinations tabs, respectively).
(XLSX)
