# Supplementary figures and images for: Dose-Dependent Regulation of Alternative Splicing by MBNL Proteins Reveals Biomarkers for Myotonic Dystrophy
Source: PLoS Genet. 2016 Sep 28;12(9):e1006316. doi: 10.1371/journal.pgen.1006316 (PMC5082313; doi:10.1371/journal.pgen.1006316)

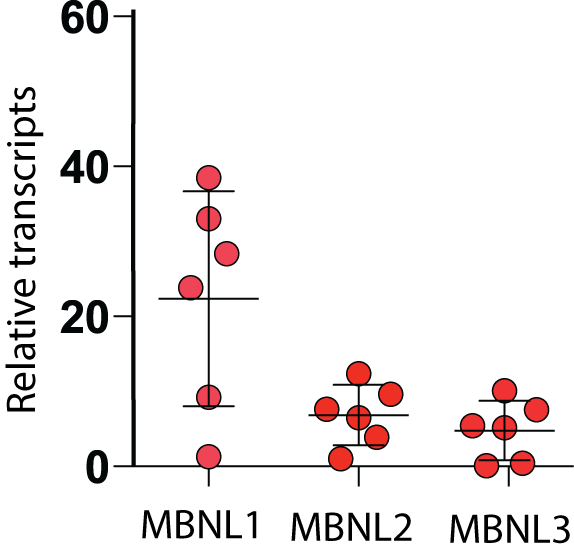

Supplement: S1 Fig — MBNL1/2/3 transcripts were estimated using RNA seq from six HEK293 cell samples. Transcripts per kilobase million are reported. (TIF) [file pgen.1006316.s001.tif]

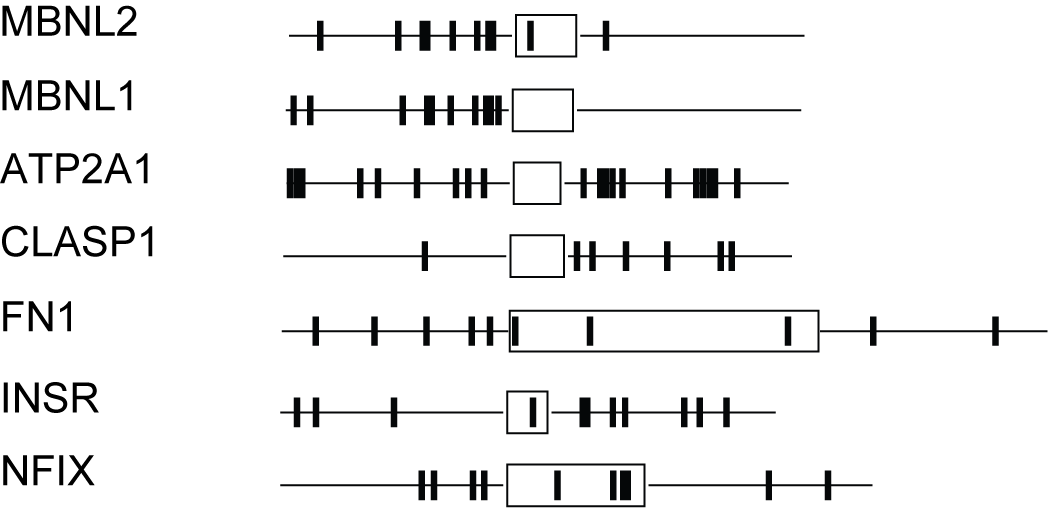

Supplement: S2 Fig — 200 nucleotides upstream and downstream of the regulated exon are depicted with YGCY motifs marked. Schematic element spacing is drawn to scale. (TIF) [file pgen.1006316.s002.tif]

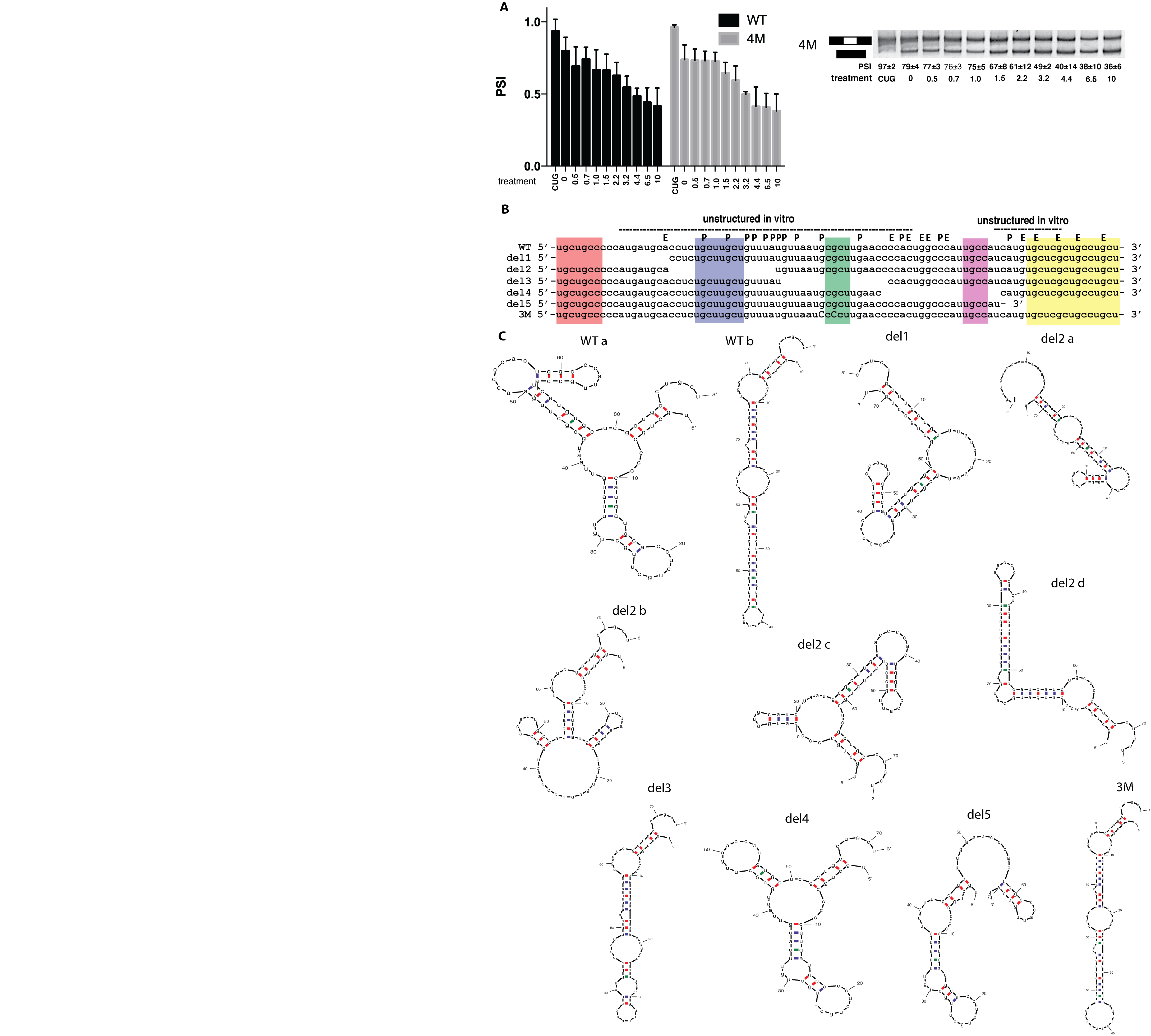

Supplement: S3 Fig — (A) 4M has similar behavior to WT. Splicing of 4M mutant compared with WT MBNL1 mini-gene reporter in triplicate. Experimental details are the same as in Fig 3. Representative gel is shown. (B) MBNL1 intron 4 is generally unstructured. Intron 4 mapped with previously determined structural data [24]. Single stranded regions are marked with a dashed line, nucleotides with increased cleavage in the presence of MBNL1 are marked with an E, and MBNL1 protected nucleotides are marked with a P (C) Predicted secondary structures obtained using the mfold server for MBNL1 WT and mutant RNAs [43]. (TIF) [file pgen.1006316.s003.tif]

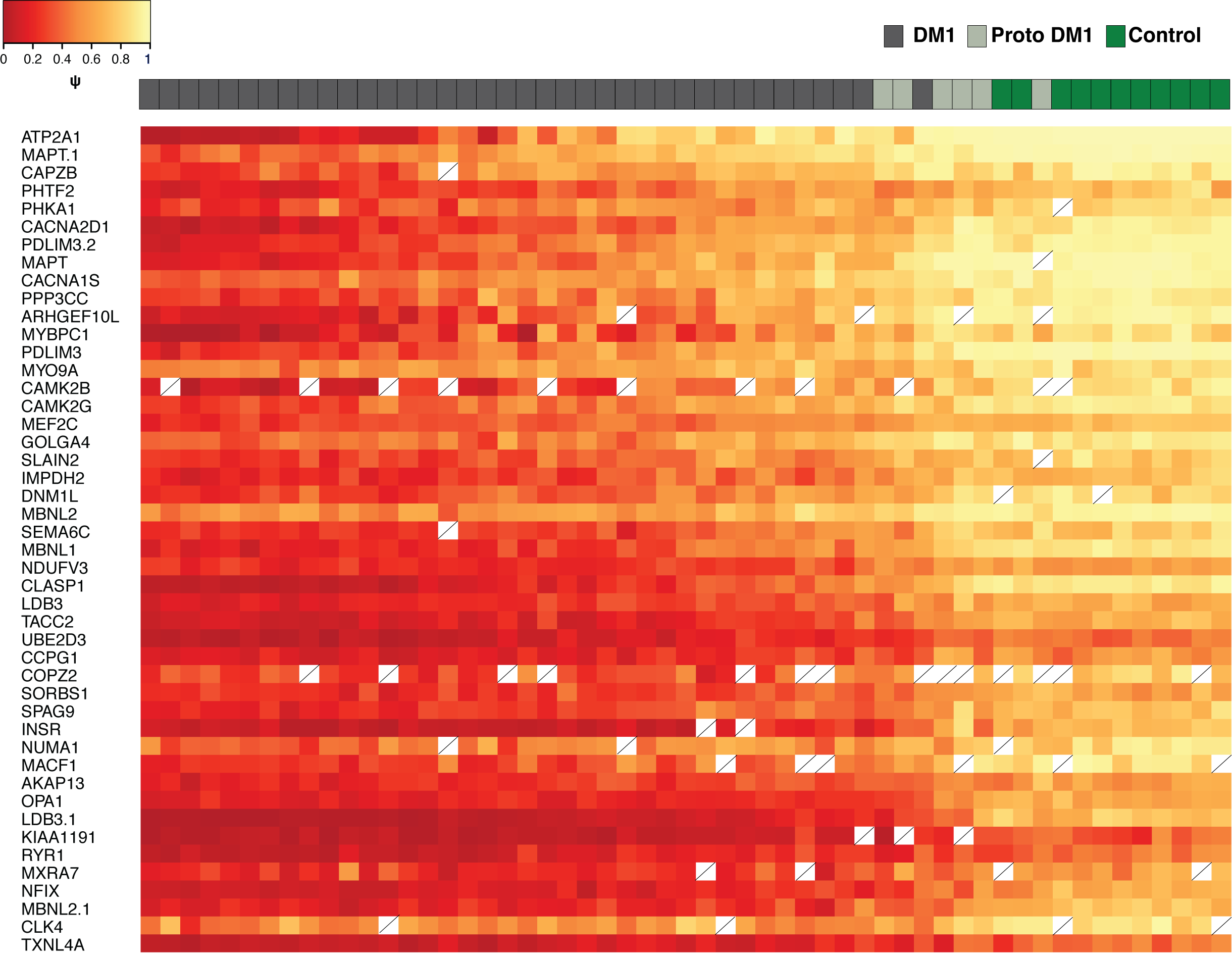

Supplement: S4 Fig — Heatmap of event Ψ estimates (un-normalized) from DM1 and control RNA-seq tibialis samples. Samples are ordered from left to right by decreasing |Δ Ψ| summed over all events. (TIF) [file pgen.1006316.s004.tif]

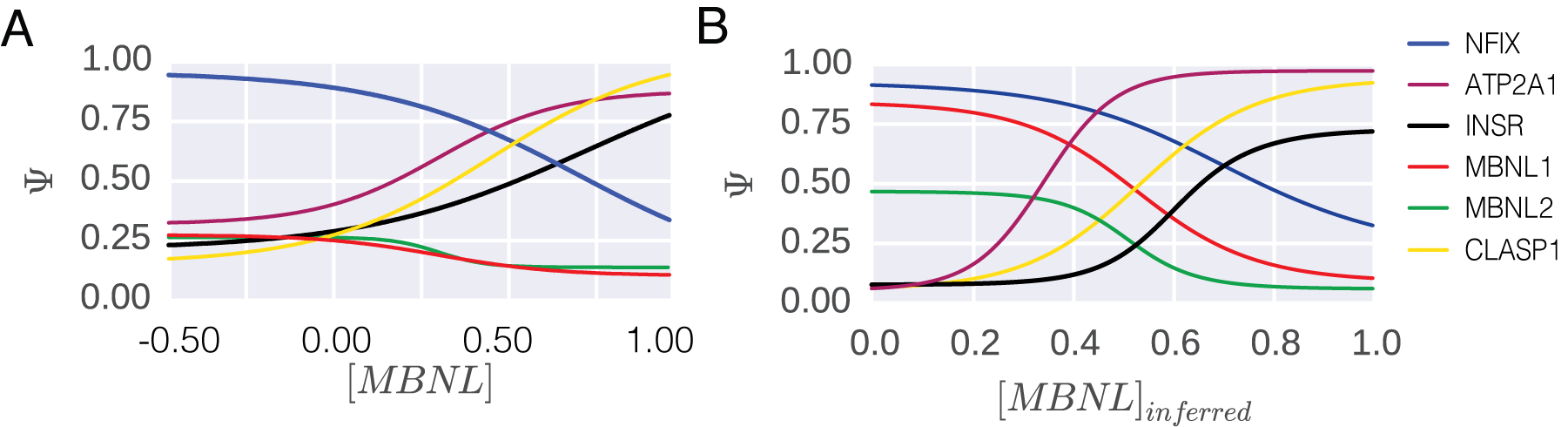

Supplement: S5 Fig — (A) Ψ was plotted against [MBNL1] as determined by western blot in HEK293 for NFIX (blue), ATP2A1 (purple), INSR (black), MBNL1 (red), MBNL2 (green), and CLASP1 (orange) (left panel) and TA muscle Ψ was plotted against inferred [MBNL] (right panel). The x-axes are log10 scale. Individual points and error for each event is shown is Fig 2 and S6 Fig. (TIF) [file pgen.1006316.s005.tif]

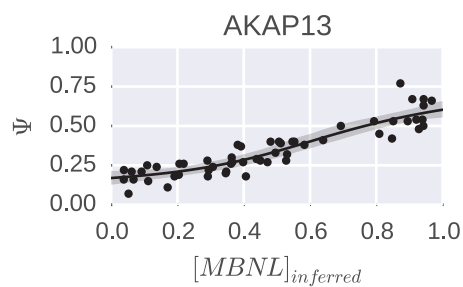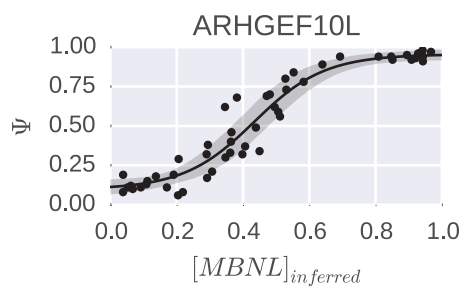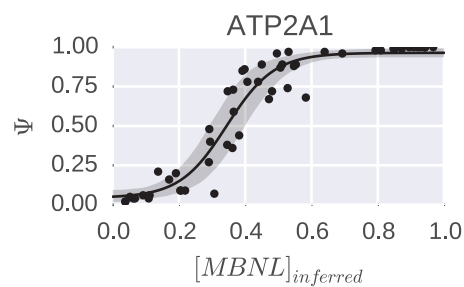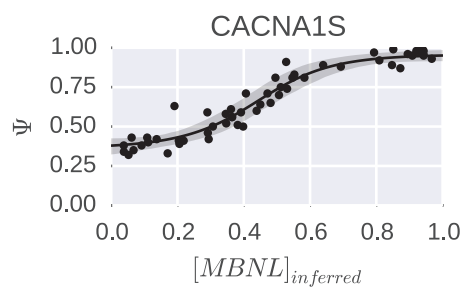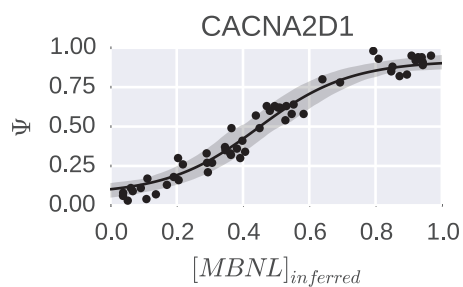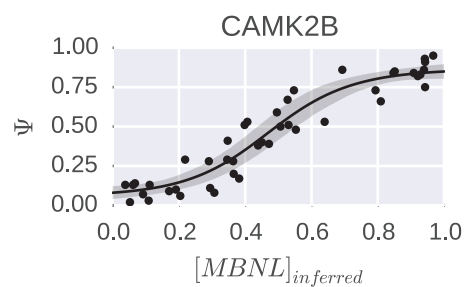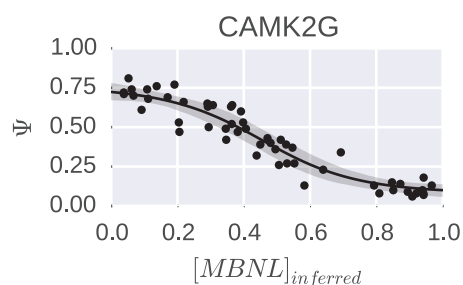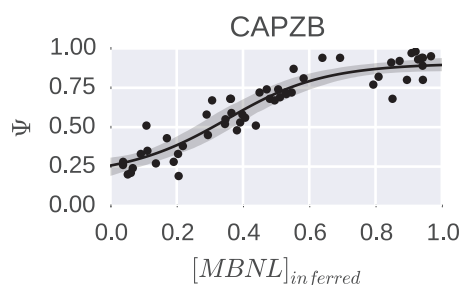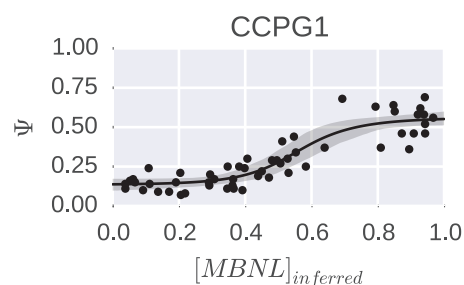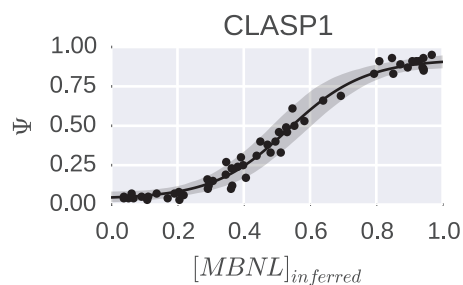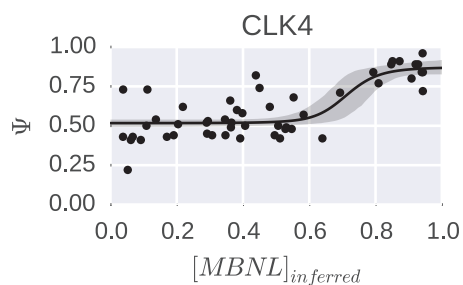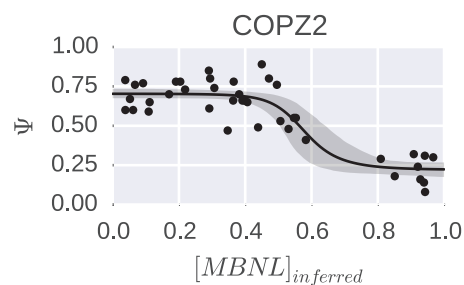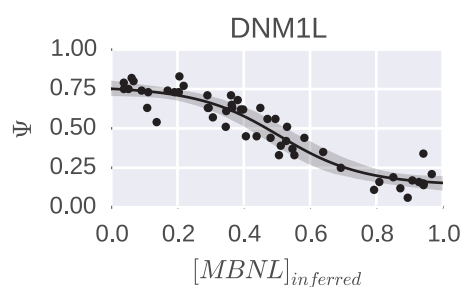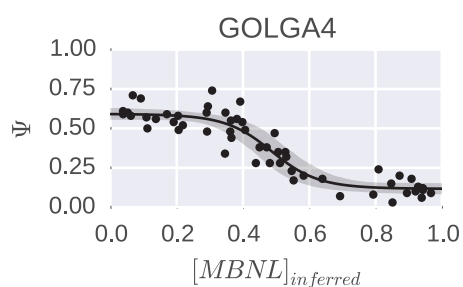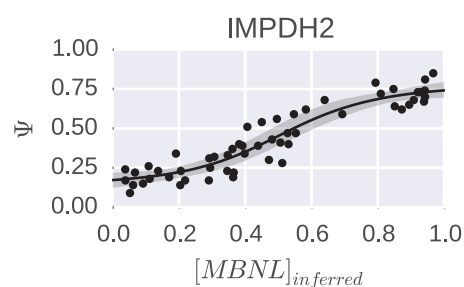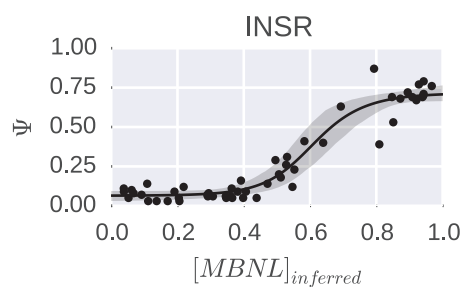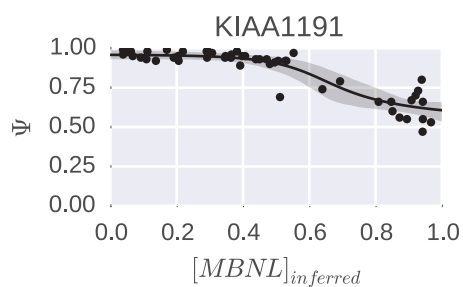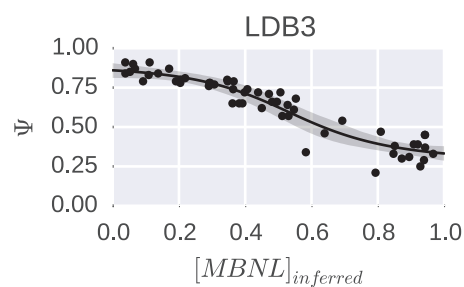

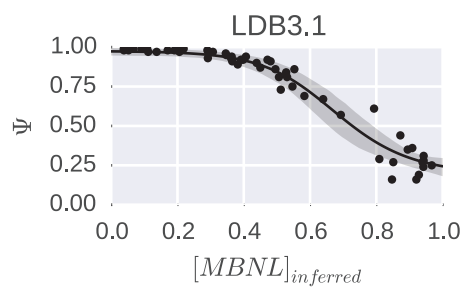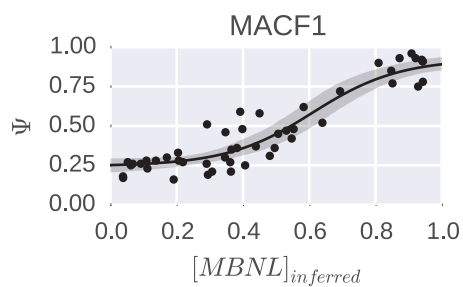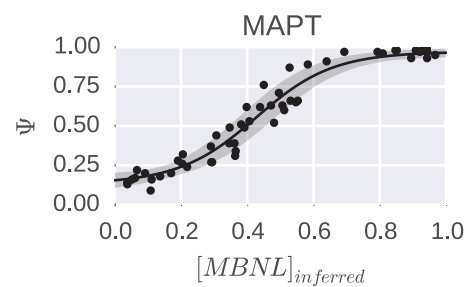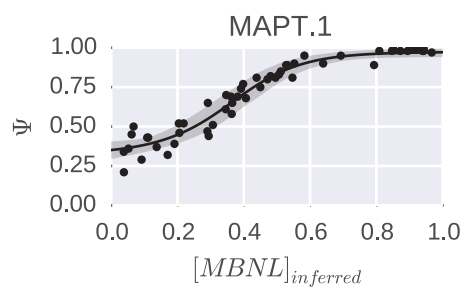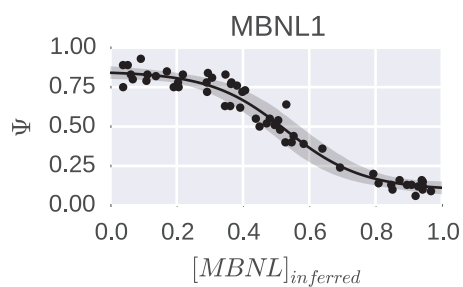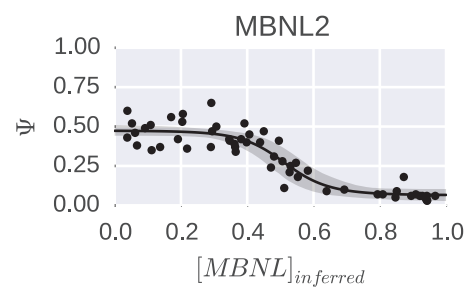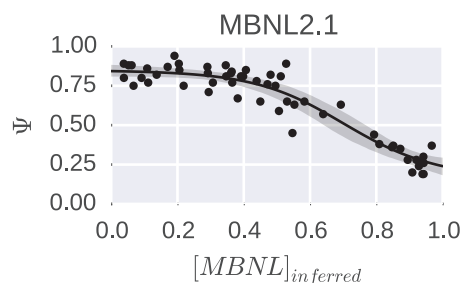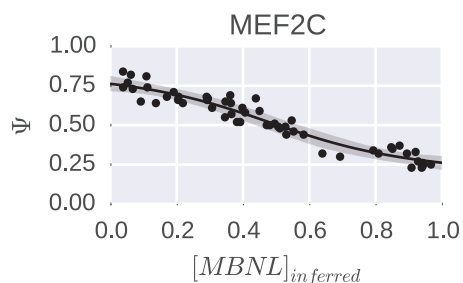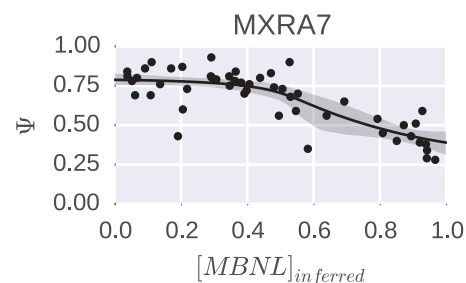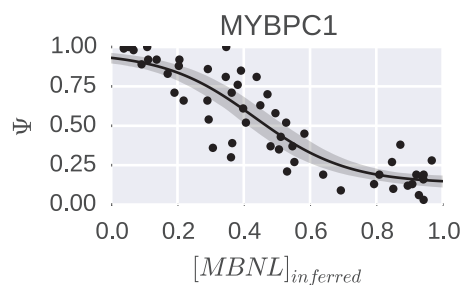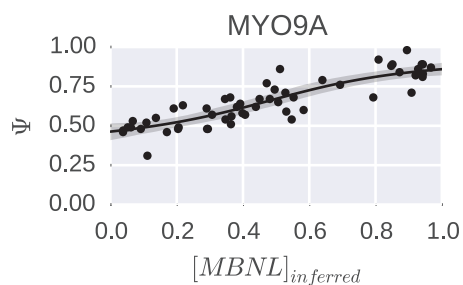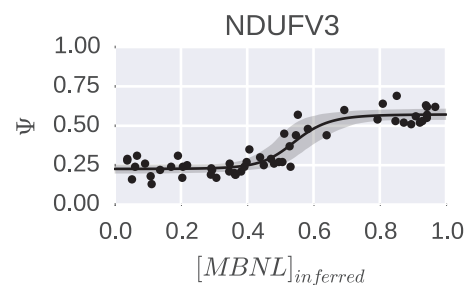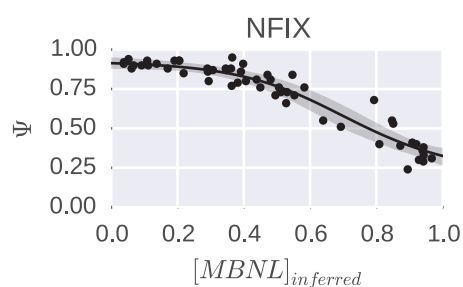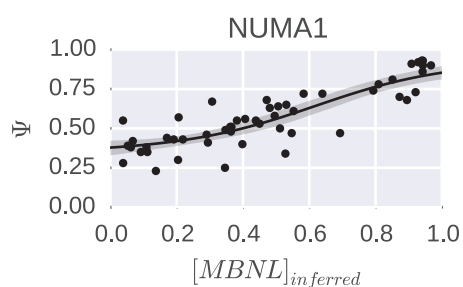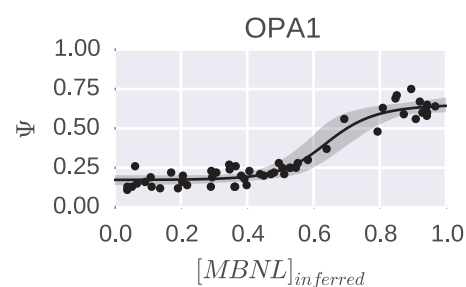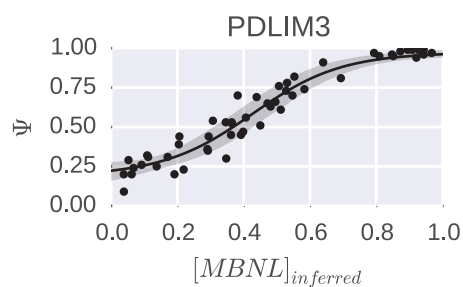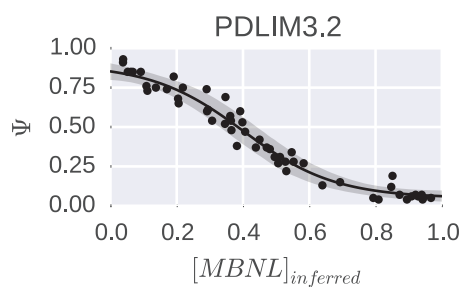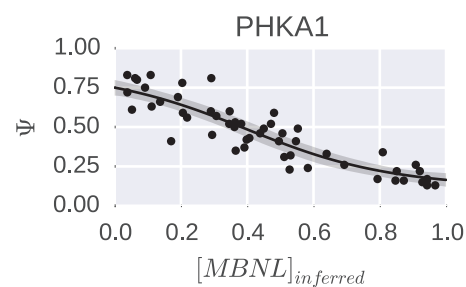

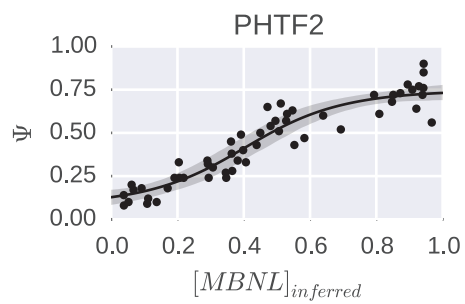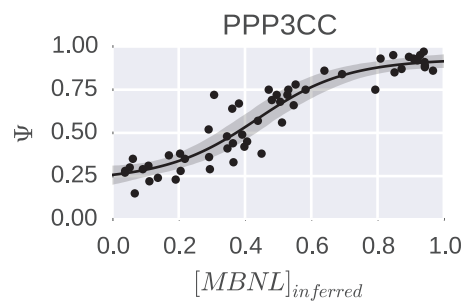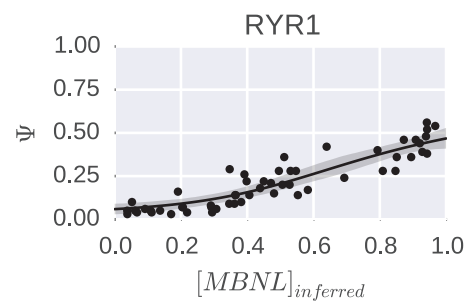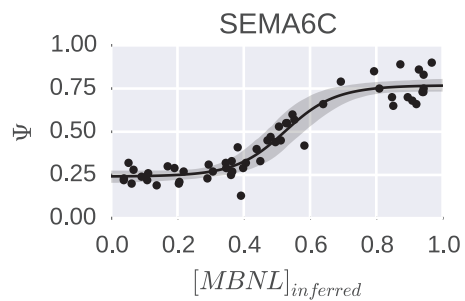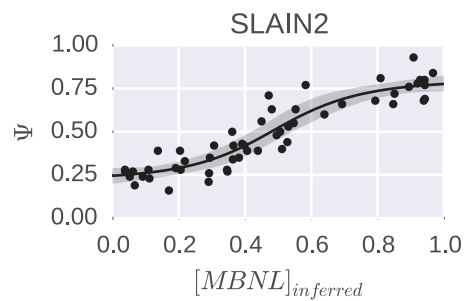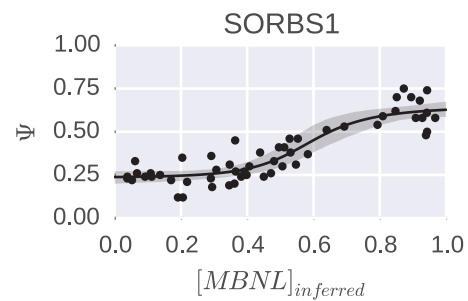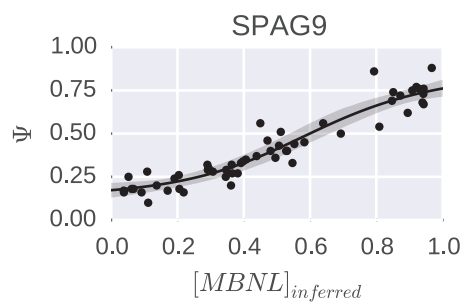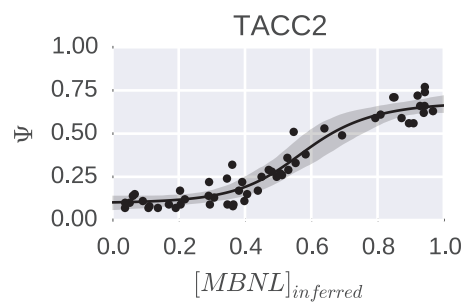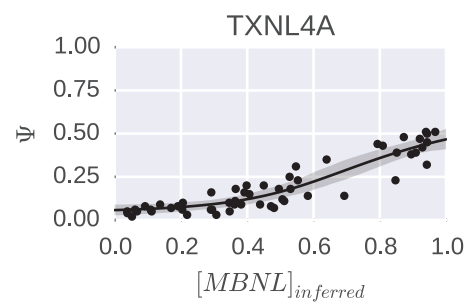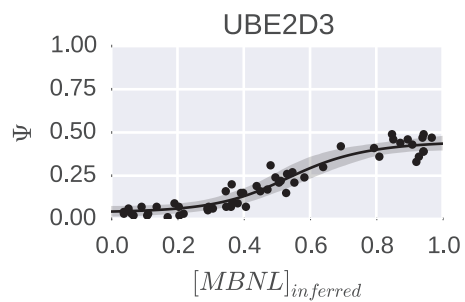

Supplement: S6 Fig — Ψ estimates are plotted against the inferred MBNL1 for each splicing event. Inferred dose-response curve are shown. (PDF) [file pgen.1006316.s006.pdf]

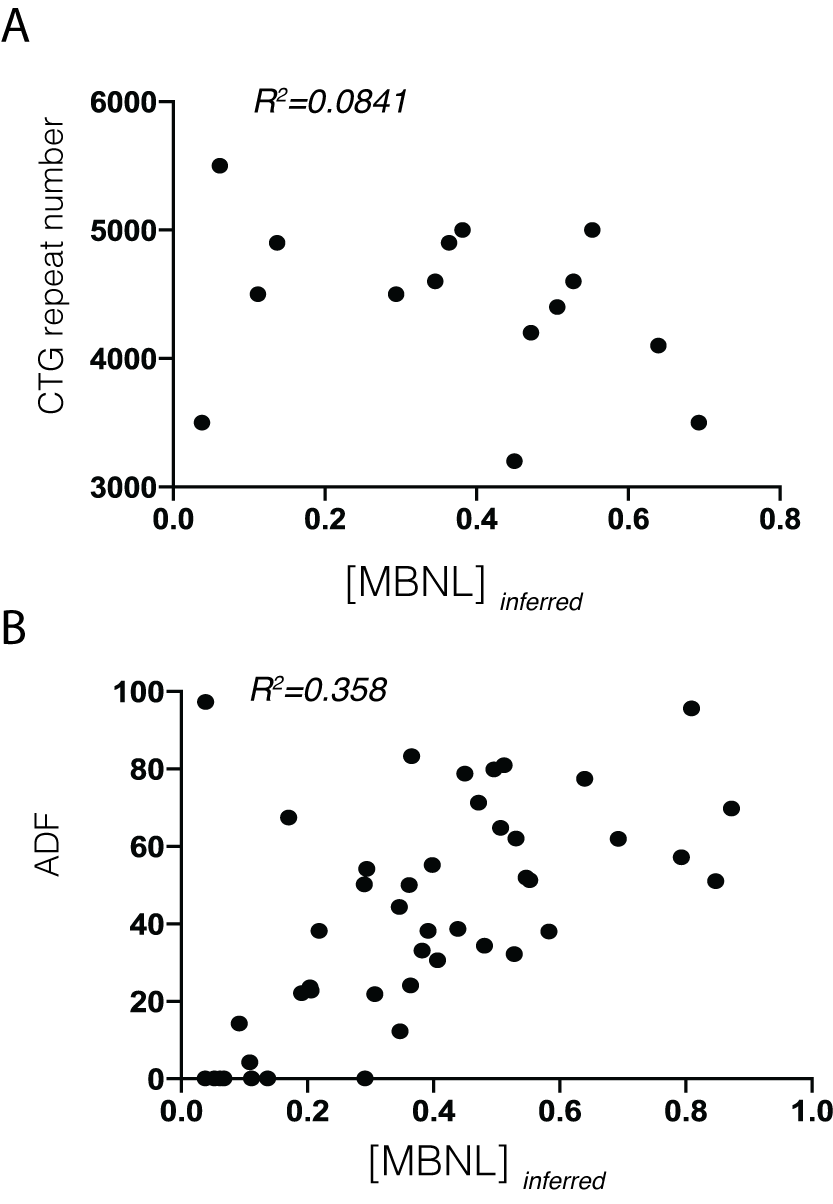

Supplement: S7 Fig — (A) CTG repeat length was not correlated with inferred [MBNL] in the tibialis muscle samples (R2 = 0.0841). (B) The maximal isometric force of ankle dorsiflexion (ADF), measurement (expressed as the percentage of strength that would be predicted in a healthy person of same age, gender, and height) moderately correlates with inferred [MBNL] (R2 = 0.358). (TIF) [file pgen.1006316.s007.tif]

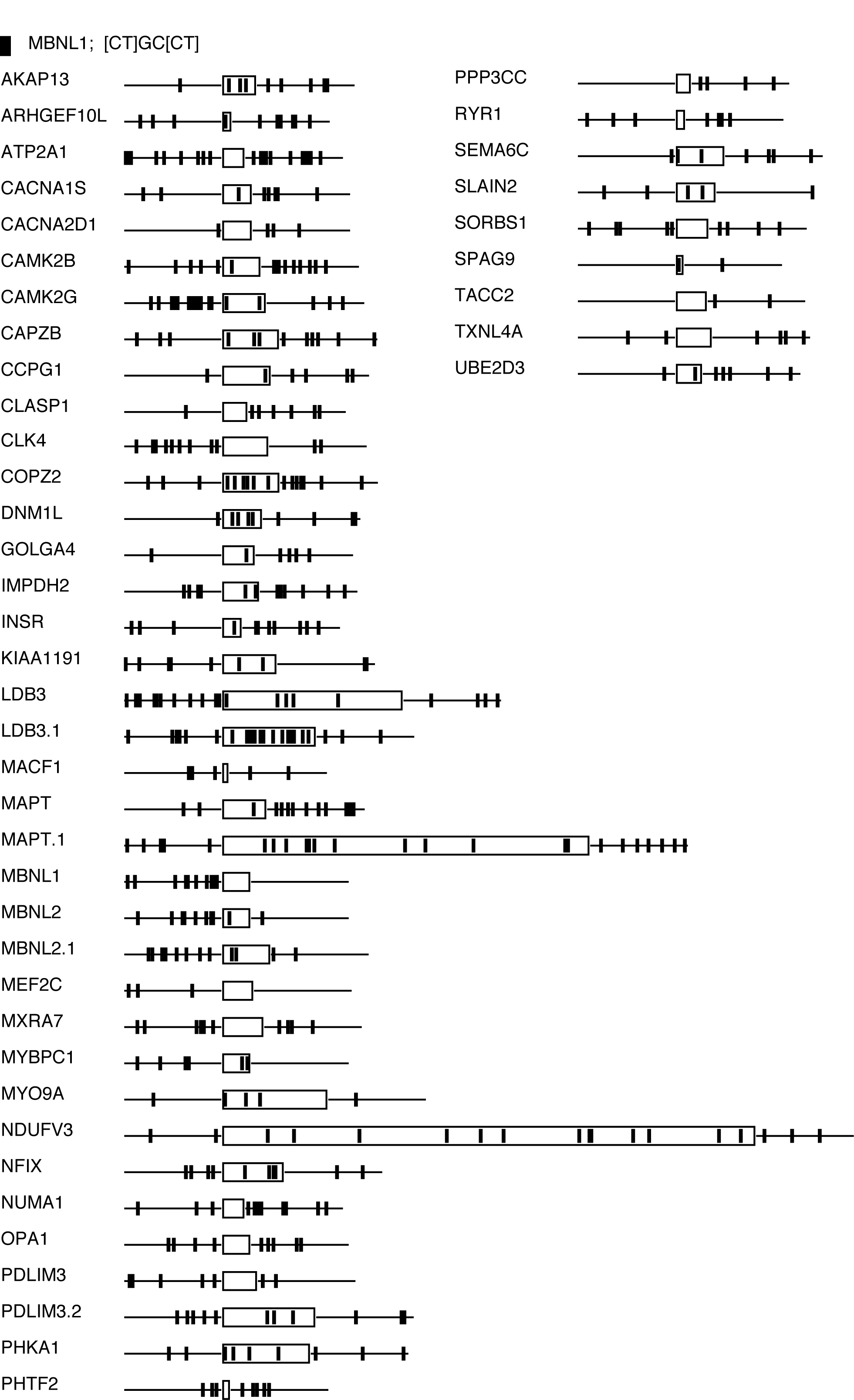

Supplement: S8 Fig — 200 nucleotides upstream and downstream of the regulated exon are depicted with YGCY motifs marker. Schematic element spacing is drawn to scale. (TIF) [file pgen.1006316.s008.tif]
